# Analysis of Gene Expression and Physiological Responses in Three Mexican Maize Landraces under Drought Stress and Recovery Irrigation

**DOI:** 10.1371/journal.pone.0007531

**Published:** 2009-10-30

**Authors:** Corina Hayano-Kanashiro, Carlos Calderón-Vázquez, Enrique Ibarra-Laclette, Luis Herrera-Estrella, June Simpson

**Affiliations:** 1 Centro de Investigación y Estudios Avanzados, Departamento de Ingeniería Genética de Plantas, Libramiento Norte Carretera Irapuato-León, Irapuato, Guanajuato, Mexico; 2 Centro de Investigación y Estudios Avanzados, Laboratorio Nacional de Genómica para la Biodiversidad (LANGEBIO), Libramiento Norte Carretera Irapuato-León, Irapuato, Guanajuato, Mexico; Purdue University, United States of America

## Abstract

**Background:**

Drought is one of the major constraints for plant productivity worldwide. Different mechanisms of drought-tolerance have been reported for several plant species including maize. However, the differences in global gene expression between drought-tolerant and susceptible genotypes and their relationship to physiological adaptations to drought are largely unknown. The study of the differences in global gene expression between tolerant and susceptible genotypes could provide important information to design more efficient breeding programs to produce maize varieties better adapted to water limiting conditions.

**Methodology/Principal Findings:**

Changes in physiological responses and gene expression patterns were studied under drought stress and recovery in three Mexican maize landraces which included two drought tolerant (Cajete criollo and Michoacán 21) and one susceptible (85-2) genotypes. Photosynthesis, stomatal conductance, soil and leaf water potentials were monitored throughout the experiment and microarray analysis was carried out on transcripts obtained at 10 and 17 days following application of stress and after recovery irrigation. The two tolerant genotypes show more drastic changes in global gene expression which correlate with different physiological mechanisms of adaptation to drought. Differences in the kinetics and number of up- and down-regulated genes were observed between the tolerant and susceptible maize genotypes, as well as differences between the two tolerant genotypes. Interestingly, the most dramatic differences between the tolerant and susceptible genotypes were observed during recovery irrigation, suggesting that the tolerant genotypes activate mechanisms that allow more efficient recovery after a severe drought.

**Conclusions/Significance:**

A correlation between levels of photosynthesis and transcription under stress was observed and differences in the number, type and expression levels of transcription factor families were also identified under drought and recovery between the three maize landraces. Gene expression analysis suggests that the drought tolerant landraces have a greater capacity to rapidly modulate more genes under drought and recovery in comparison to the susceptible landrace. Modulation of a greater number of differentially expressed genes of different TF gene families is an important characteristic of the tolerant genotypes. Finally, important differences were also noted between the tolerant landraces that underlie different mechanisms of achieving tolerance.

## Introduction

Abiotic stress is a major limiting factor for plant growth and food production in many regions of the world and its effects will become more severe as desertification claims more of the world's arable land. Among environmental stresses, drought has the greatest effect on agriculture worldwide [1], affecting more than one-fifth of the tropical and subtropical areas used for maize production [2]. As an example, in Mexico around 80% of all maize cultivated is grown under rain-fed conditions [3], where the possibilities for alleviating water stress are limited [2]. Therefore, an urgent need exists to develop drought-tolerant varieties either by conventional breeding or by genetic engineering in order to cope with the rising demand for maize to feed both humans and animals.

Due to a unique genome structure and continuous human selection for over 7000 years, maize is one of the most plastic plant species in terms of its adaptation to different environmental conditions, capable of growing at high and low altitudes and in tropical, subtropical and temperate climates. This genetic variability has been exploited to produce locally adapted drought tolerant maize cultivars for the dry tropical areas of Indonesia, Kenya, Mexico and Colombia [4]. Currently marker-assisted selection (MAS) is used in the development of maize germplasm with improved stress tolerance [5] based on QTL's affecting root architecture, leaf ABA concentration and other drought-related traits [6]. Despite these efforts, improvement programs for drought stress in maize have advanced slowly and substantial research is needed to adapt the improved genetic materials to particular environmental conditions [4], where they should not only withstand greater levels of drought but also perform well under optimal conditions [2]. Moreover, local landrace accessions could provide novel alleles that will complement strategies based on existing stress-adaptation mechanisms [7].

During evolution, plants have acquired a myriad of developmental and metabolic strategies to optimize water uptake and efficiently balance this with water utilization during vegetative growth and reproduction [8], making drought tolerance a complex multigenic trait. In the past decade, research to unravel the molecular processes involved in drought tolerance has received special attention [9], for reviews see [10], [11], [12]. Physiological studies have shown that sugars, sugar alcohols, amino acids and amines function as osmolytes, protecting cellular functions from the effects of dehydration and are known to accumulate under drought stress conditions in different plant species [13]. Reduction in vegetative growth, stomatal closure and a decrease in the rate of photosynthesis [14] are among the earliest responses to drought, protecting the plant from extensive water loss [9].

More recently, genomic technologies have provided high-throughput integrated approaches [15] to investigate global gene expression responses not only to drought but also to other abiotic stresses [9]. Microarray profiling under drought stress has been carried out in different plant species such as *Arabidopsis* [16], [17], [18], rice [19], barley [20], [21] and wheat [22]. These studies identified differentially expressed transcripts of genes involved in photosynthesis, ABA synthesis and signaling, biosynthesis of osmoprotectants, protein stability and protection, reactive oxygen detoxification, water uptake and a myriad of transcription factors including several members of the zinc finger, WRKY, and bZIP families. To date gene expression studies in maize in response to water stress have investigated different organs such as roots [23] and developing kernels [24] or particular developmental stages [25]. However, no reports have addressed comparisons between the drought stress responses of susceptible and tolerant maize genotypes or genotypes that have been reported to possess different tolerance mechanisms.

Mexican maize genotypes with apparently different mechanisms for achieving drought tolerance have been reported. For instance, Cajete Criollo (CC), cultivated mainly in Oaxaca State, Mexico, has a high tolerance to low water content in the soil and a long vegetative cycle with slow growth until the rains arrive when a rapid response in terms of growth and recovery occurs [26]. Michoacán 21 (M21) from the Purépecha highlands in Michoacán State (Mexico) was described as a landrace with a clear response to drought and cold stress [27]. The mechanism of tolerance of M21 was termed “latency” and consists of prolonging the vegetative stage under drought stress without flowering and a rapid return to normal growth and completion of the reproductive cycle even when the rains begin. M21 is more resistant to permanent wilting in seedlings in comparison to other maize genotypes and has a higher transpiration rate under well irrigated conditions as compared to conditions of limiting water resources [27].

The aim of this study was to analyze the differences in physiological responses and gene expression of one susceptible (85-2) and two drought-tolerant (Cajete criollo, CC and Michoacán 21, M21) maize landraces. The 3 genotypes were subjected to intermediate (10 days without water) and severe (17 days without water) drought stress treatments followed by recovery irrigation and global gene expression were evaluated at the different time points using a 56K oligonucleotide maize microarray. The results confirm that different physiological responses and different gene expression patterns occur under drought stress and recovery in the 2 tolerant genotypes, and provide insights as to how changes in gene expression could lead to drought tolerance and recovery in maize. Expression patterns of genes involved in photosynthesis and carbohydrate and proline metabolism, those encoding transcription factors and those known to be involved in other abiotic stress responses were studied in more detail, providing information on the correlation between the physiological and gene expression responses of the three genotypes, and allowing the identification of specific genes and expression patterns associated with particular metabolic pathways in each of the 3 landraces.

## Results

### Physiological effects of drought stress

Changes in leaf and soil water potentials during drought stress treatments

**Figure 1:**
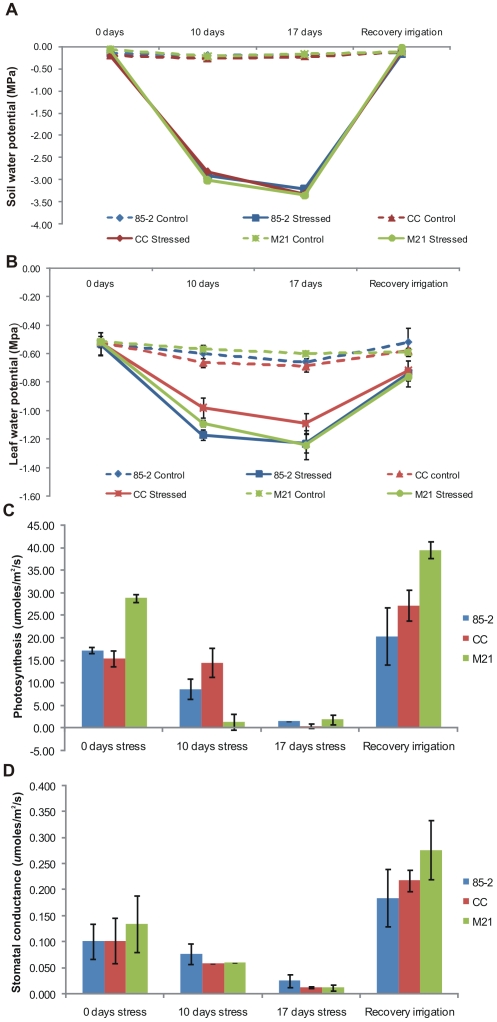
Physiological parameters of maize plants under drought stress and recovery irrigation. (A) Soil water potential, (B) Leaf water potential, (C) Photosynthetic rate, (D) stomatal conductance. Data are from three measurements from different samples with standard error.

To ensure that plants were grown under the required drought stress conditions, soil and leaf water potentials were monitored throughout the experiment. As shown in Figure 1A, soil water potentials (ψ_s_) were similar for all genotypes throughout the experiment. To determine whether plant water status differed between the three genotypes, leaf water potentials (ψ_l_) were monitored at 10 and 17 days of drought stress and after recovery irrigation. Irrigated plants of all three genotypes maintained relatively constant levels of leaf water potential (ψ_l_) of between −0.52 to −0.53 MPa (Figure 1B) throughout the experiment. Leaf water potentials (ψ_l_) in stressed plants became more negative as the level of stress increased, −0.98 to −1.17 MPa after 10 day of stress and –1.06 to −1.23 MPa for 17 days of stress. At 10 days stress, the two drought tolerant genotypes showed slightly higher levels of leaf water potential as compared to the susceptible genotype. At 17 days stress, 85-2 and M21 showed a similar decrease in water potential, whereas CC still maintained higher leaf water potential. Ten hours after the recovery irrigation, all three genotypes showed a similar increase in water potential, to levels only slightly lower than before the drought stress treatment.

### Rates of photosynthesis

To determine the effect of drought on photosynthetic activity in the three genotypes, rates of photosynthesis were determined at 10 and 17 days of drought stress and 12 h after the recovery irrigation. Under well-watered conditions, M21 showed the highest photosynthetic rate followed by CC and 85-2. Drought stress treatments caused reductions in rates of photosynthesis in all three landraces. M21 showed the most rapid reduction of photosynthetic rate, dropping by 77.5% after 10 days and 86.92% after 17 days stress in comparison to the levels in irrigated plants. CC showed the slowest decrease in photosynthetic rate, decreasing by only 30.55% after 10 days of drought and by 46% after 17 days of drought, whereas 85-2 showed a reduction of 52.2% and 88.4% after 10 and 17 days of drought stress, respectively (Figure 1C). Whereas M21 showed the fastest reduction in photosynthesis rate during drought treatment and a more rapid recovery of photosynthetic activity upon recovery irrigation, CC appeared to maintain a higher rate of photosynthesis during drought and showed a slower recovery on irrigation. 85-2 showed an increase in rate of photosynthesis following the recovery irrigation but to a lesser extent than M21 (Figure 1C).

### Stomatal conductance

Drought stress also caused a gradual decrease in stomatal conductance in all three landraces (Figure 1D) as stress became more severe. Values for irrigated plants before stress were similar for all three landraces, although M21 showed a slightly higher value. At 10 days of drought stress, M21 showed a sharp drop (76.92%) in stomatal conductance compared to the irrigated plants, whereas for CC and 85-2 stomatal conductance decreased only 20% and 14.3% respectively. At day 17 under drought stress all three landraces showed a significant decrease in stomatal conductance (85.71% for 85-2, 90% for CC and 84.61% for M21) as compared to the corresponding value prior to the stress treatment. Upon recovery irrigation all three landraces showed a rapid increase in stomatal conductance of 94.12%, 93.33% and 88.89% (for 85-2, CC and M21 respectively), greater than the corresponding value prior to the stress treatment (Figure 1D).

### Variation in sugar concentration

**Figure 2:**
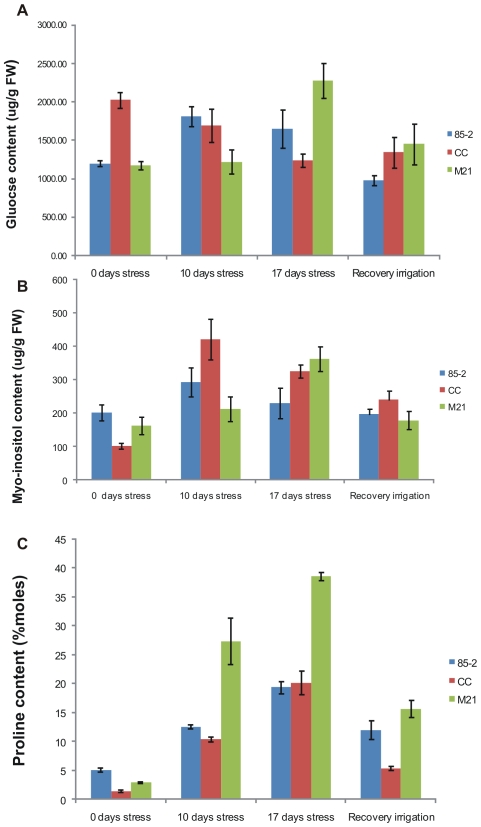
Sugar and proline content under 10 and 17 days of drought stress and recovery irrigation. (A) Glucose content, (B) Myo-inositol content, (C) Proline content. Data are the means of three different samples with standard error.

Analysis of glucose and myo-inositol content (Figures 2A and 2B) indicated a rise in both these sugars to a peak at 17 days stress in M21, whereas CC showed a drop in glucose levels as drought stress progressed and a peak in myo-inositol levels at 10 days stress. Landrace 85-2 showed the highest levels for both sugars at 10 days stress. Sucrose levels were maintained relatively constant in all 3 landraces throughout the drought experiment with a slight rise to a maximum at 17 days stress in all cases (data not shown).

### Proline content

Drought stress causes changes in amino acid metabolism in general and in particular accumulation of proline has been correlated with osmoprotection in several plant species [9], [15]. Determination of proline levels in the 3 landraces under drought stress and recovery showed a 2.5 fold increase at 10 days stress in 85-2 and a 7–9 fold increase in CC and M21. At 17 days stress, proline content in 85-2 had increased by 4 fold and by approximately 14 fold in CC and M21. However, M21 was the landrace with greatest proline accumulation. On recovery irrigation proline levels fell in all landraces to 2 fold higher than prior to drought stress in 85-2 and around 5 fold greater in CC and M21 (Figure 2C). These results suggest that osmoprotection by proline accumulation may be an important factor in achieving tolerance which is shared by both tolerant landraces.

### Transcription profiling

**Figure 3:**
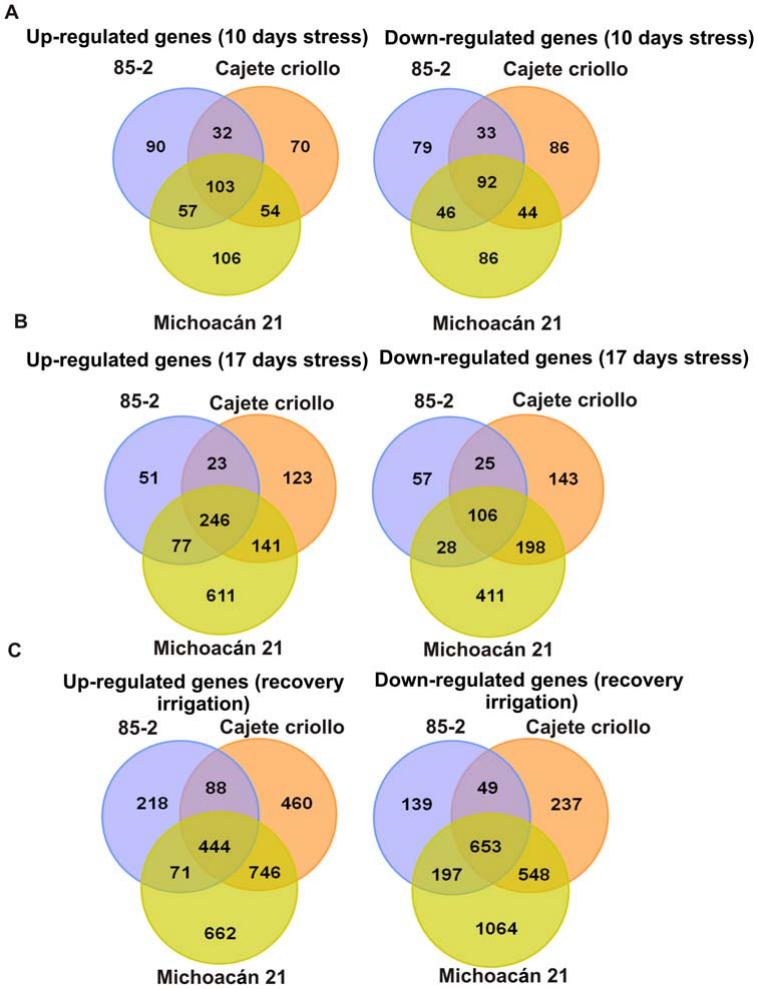
Venn diagrams of up- and down-regulated transcripts under drought stress and on recovery irrigation. (A) Differentially expressed genes at 10 days stress, (B) Differentially expressed genes at 17 days stress, (C) Differentially expressed genes at recovery irrigation. Number of genes with at least 2 fold change and FDR ≤0.5 are shown for each landrace identified by the name above the circle.

The Maize Oligonucleotide Array (MOA) was used to analyze the differences in gene expression under drought stress between the three maize landraces. A numerical comparison of differentially expressed transcripts between the three genotypes under different drought stress treatments is shown in Figures 3A and 3B. Differences in number, level of expression and type of responsive genes can be seen between the different landraces and under the different drought stress treatments. In general, changes between stress treatments and untreated plants (both up and down-regulated) were greatest for M21. CC showed an intermediate response and 85-2 the lowest number of differentially expressed genes. Throughout the text all the differences in the numbers of up or down regulated genes between the three landraces are statistically significant as determined by chi^2^ analysis (Table S12) unless otherwise stated. Although certain genes were differentially expressed in all three landraces many were specific to each landrace.

At 10 days drought stress, 103 up-regulated and 92 down-regulated genes were common to the three landraces. M21 showed a higher number of specific, differentially expressed transcripts (106 up and 86 down regulated) compared to 85-2 (90 up- and 79 down regulated) and CC (70 up and 86 down-regulated genes) as seen in Figure 3A. As the level of drought stress increased, the number of differentially expressed genes also increased. At 17 days stress, a total of 246 up-regulated and 106 down-regulated genes were common to all three landraces. At the same time point, the susceptible landrace (85-2) showed the lowest number (51 up- and 57 down-regulated), CC showed an intermediate number (123 up- and 143 down-regulated genes) and M21 the highest number (611 up and 411 down-regulated genes) of specific differentially expressed genes (Figure 3B). At 17 days of stress, the number of differential transcripts shared by the tolerant landraces (141 up and 198 down-regulated) was also greater than those shared between either of the tolerant landraces and the susceptible landrace.

In total, a greater number of differentially expressed transcripts were observed upon recovery irrigation in comparison to those found at 10 and 17 days of stress (Figure 3C). Under these conditions 444 transcripts were up- and 653 down-regulated common in all three landraces. A similar pattern of expression to that observed at 17 days stress was also observed at recovery irrigation, with 85-2 showing the lowest level (218 up and 139 down-regulated), CC intermediate (460 up and 237 down-regulated) and M21 the highest level (662 up and 1064 down-regulated) of genotype specific differentially expressed genes. The number of differentially expressed transcripts shared by the tolerant landraces (746 up and 548 down-regulated) was also higher than those shared between the tolerant landraces and 85-2. Moreover, we found that 65.49% of up-regulated genes at 17 days stress were repressed on recovery; whereas 55.68% of the genes repressed at 17 days stress were induced at recovery. We also found that 8.13% of the genes were induced both at 10 and 17 days stress.

The differential expression pattern observed in the microarray experiment was evaluated for 16 genes using qRT-PCR (Figure S1). Most of the genes showed the same expression pattern in both the microarray experiment and the qRT-PCR analysis. Differences were mainly observed at the quantitative level, with the qRT-PCR analysis showing in general a higher fold of induction or repression than the microarray analysis. Similar quantitative differences between qRT-PCR and microarray data have been reported previously [28] and [29].

### Functional classification of differentially expressed transcripts

**Figure 4:**
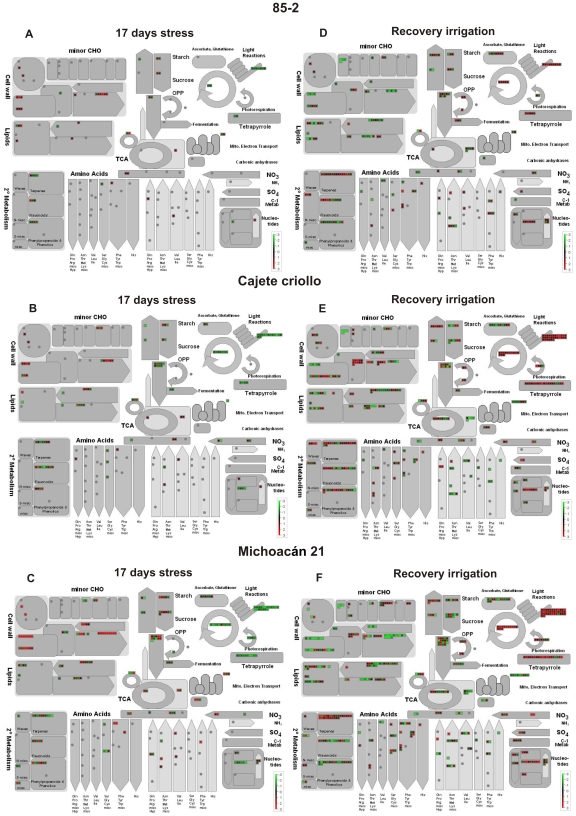
Overview of differentially expressed transcripts involved in different metabolic processes under stress and recovery irrigation. (A) Genes at 17 days stress in 85-2, (B) Genes at 17 days stress in CC, (C) Genes at 17 days stress in M21, (D) Genes at RI in 85-2, (E) genes at RI in CC, (F) Genes at RI in M21. Gene transcripts that are induced or repressed are shown in red or green coloring respectively as shown in the color bar in each panel. The MapMan sotware was used to show the different functional categories involved. (CC: Cajete criollo, M21: Michoacán 21).

Due to the limited functional annotation currently available for maize transcripts, functional classification of differentially expressed transcripts was carried out using the MapMan hierarchical ontology software [30] and BioMaps at the Virtual-Plant site (www.virtual plant.org) as described in Materials and Methods with similar results. However, the analyses presented here were based on MapMan software. A general overview of the metabolic and cellular processes, for which differentially expressed genes were identified, are shown in Figures 4A, 4B and 4C for 85-2, CC and M21 respectively for 17 days stress and Figures 4D, 4E and 4F for 85-2, CC and M21 respectively for recovery irrigation. These global maps of differentially expressed genes illustrate that the tolerant genotypes showed more wide-ranging metabolic and cellular responses during drought stress and recovery irrigation than 85-2. The microarray data using BioMaps are shown in Table S4, Table S5, Table S6, Table S7, Table S8, Table S9, Table S10 and Table S11.

### Photosynthesis and carbohydrate metabolism

**Figure 5:**
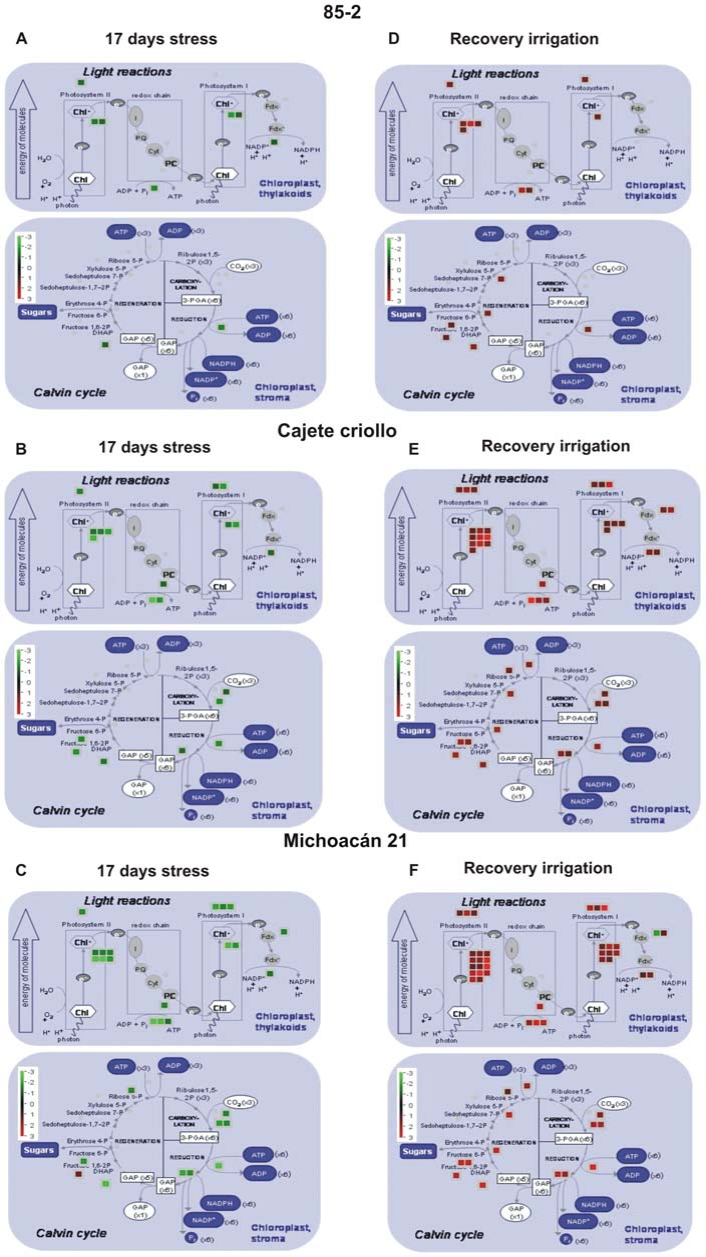
Differential expression of genes involved in photosynthesis under drought stress and at recovery irrigation. (A) Genes differentially expressed at 17 days stress in 85-2, (B) Genes differentially expressed at 17 days stress in CC, (C) Genes differentially expressed at 17 days stress in M21, (D) Genes differentially expressed at RI in 85-2, (E) Genes differentially expressed at RI in CC, (F) Genes differentially expressed at RI in M21. Gene transcripts that are induced or repressed are shown in red or green colouring respectively as shown in the color bar in each panel. (CC: Cajete criollo, M21: Michoacán 21).

To determine whether the observed changes in rates of photosynthesis described above correlated with changes in gene expression, the effect of drought stress and recovery irrigation on the expression of genes encoding components of the photosynthetic machinery was analyzed in detail. At 10 days stress 4, 1 and 2 up-regulated (differences not statistically significant) and 13, 17 and 22 down-regulated genes related to photosynthesis were found in 85-2, CC and M21 respectively (Table S1). At 17 days stress, all differentially expressed photosynthesis related genes were down regulated in all three genotypes (85-2: 11, CC: 28 and M21: 45, Table S2). The only notable exception was a transcript for a putative fructose-bisphosphate aldolase which was up-regulated in M21. A general view of this data is shown in Figures 5A, 5B and 5C for one representative member of each photosynthesis-related gene family. Interestingly, CC and M21 showed more down-regulated gene families than 85-2. For instance, 3 Calvin cycle-related genes were down-regulated in 85-2 as compared to 7 in CC and 12 in M21. Down-regulated transcripts of Calvin cycle genes such as triosephosphate isomerase (TPI), fructose-1,6-bisphosphatase (FBPase), Rbcs (RuBisCO small subunit) and Rubisco activase were identified.

On recovery irrigation the pattern of expression was reversed, with an increase in differential expression of photosynthesis related genes in all three genotypes. The responses of the landraces were low (17), intermediate (61) and high (81) in terms of numbers and levels of up-regulation of specific genes for 85-2, CC and M21 respectively (Figures 5D, 5E and 5F). With respect to Calvin cycle-related genes 6, 19 and 21 were up-regulated in 85-2, CC and M21 respectively.

Sugar metabolism is closely linked to photosynthesis and differences in accumulation of glucose and myo-inositol between the genotypes were observed as described above. In this context, 8, 15 and 28 genes related to carbohydrate metabolism were found to be up-regulated on recovery irrigation in 85-2, CC and M21 respectively. A transcript for β-amylase is induced in all 3 genotypes at 10 and 17 days stress and to a much greater extent in M21 at 17 days stress (Table S2) whereas on recovery irrigation three transcripts for β-amylase were repressed in M21 (Table S3). Two transcripts for hexokinases (HXK) were found to be up-regulated at 17 days stress in M21 and one in 85-2 whereas in CC they did not reach the 2 fold level (1.84 and 1.4 fold change). On recovery three hexokinase transcripts were repressed in M21, but in 85-2 constitutive expression was observed whereas in CC a 0.64 fold change was observed; suggesting that changes occur in glucose metabolism under stress that are then reversed or repressed on recovery. Perhaps surprisingly, the genes encoding enzymes involved in synthesis of myo-inositol (Ins (3) P synthase and MI monophosphatase) are repressed or remained constant under stress in all 3 genotypes in spite of the fluctuations in myo-inositol levels described above. On recovery, however, M21 shows a slight increase in the expression of these genes.

Induction and repression of genes associated with proteins involved in HXK dependent and independent signaling pathways as proposed for *A. thaliana* was also observed. For example genes associated with HXK dependant glucose signaling such as CAB and Rbcs were repressed under stress but induced on recovery, whereas PLD was induced under drought and repressed on recovery for all 3 landraces. For the HXK independent pathway, AGPase and PAL were all down regulated or constitutive in CC and 85-2 but up-regulated in M21 at 17 days stress, while all were down regulated or constitutive in all 3 landraces on recovery (Table S2 and Table S3).

Genes associated with sucrose metabolism were mainly constitutive or repressed at 17 days stress; however, we found 1 and 4 transcripts up-regulated for sucrose synthase (Susy) in CC and M21 respectively at 17 days stress. On recovery, most of the genes related to sucrose metabolism were induced in CC and M21, whereas levels of expression of these genes in 85-2 remained constant.

### Aminoacid metabolism

#### Proline metabolism

The expression patterns of genes encoding enzymes involved in proline synthesis and degradation agreed well with the levels of proline determined. A pyrroline-5-carboxylate synthase gene was up regulated under stress in CC and constitutive in M21 and 85-2 under 17 days stress. In contrast an ornithine aminotransferase involved in a different proline biosynthetic pathway was up-regulated specifically in M21 under stress (Table S2). This may indicate preferential use of one or the other proline biosynthetic pathways in the tolerant landraces under drought stress. A transcript for proline oxidase involved in proline degradation showed a slight decrease (0.95 and 0.58 fold) in CC and M21 respectively under drought but was up-regulated in 85-2. On recovery irrigation, a transcript for pyrroline-5-carboxylate dehydrogenase (P5CDH) involved in proline degradation was up-regulated in the tolerant landraces but constitutive in 85-2. Two transcripts for proline oxidase were up-regulated in CC and one in 85-2 (Table S3) during recovery irrigation, no changes were detected in M21. On the other hand, a gene encoding pyrroline-5-carboxylate synthetase (P5CS), involved in proline synthesis, was down-regulated under recovery irrigation in M21, although 85-2 and CC showed 0.79 and 0.54 fold changes respectively.

### Signaling and abiotic stress related genes

**Figure 6:**
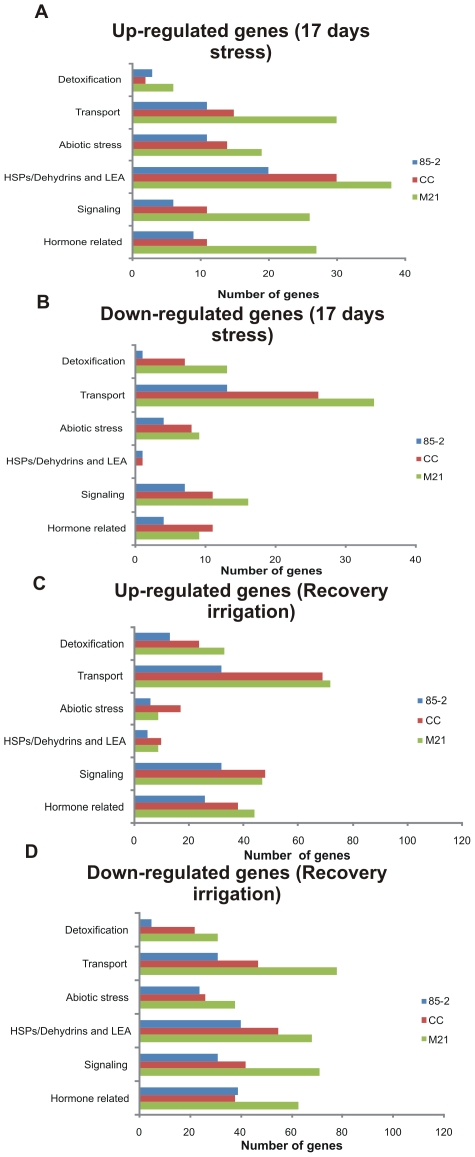
Functional classification of abiotic stress genes under 17 days of drought stress and recovery irrigation. (A): Up-regulated genes under 17 days of drought stress, (B): Down-regulated genes under 17 days of drought stress, (C): Up-regulated genes under recovery irrigation, (D): Down-regulated genes under recovery irrigation.

Metabolic responses to different abiotic stresses are often shared; therefore in order to compare changes in expression of previously characterized stress related genes between the three landraces under drought stress, functionally annotated transcripts were grouped into different categories: hormone metabolism, signaling, transport, detoxification, heat-shock proteins (including dehydrins and LEA) and abiotic stress genes. In general, fewer stress-related genes were differentially expressed at 10 days as compared to 17 days stress. Although similar patterns of up and down regulation were observed for all categories in all three landraces, M21 showed the highest changes in transcript abundance of differentially expressed genes in all categories and many differentially expressed genes were unique to M21 (Table S3). On recovery irrigation, in general the number of induced genes in each category was lower than those that were repressed. CC and M21 showed very similar patterns of up-regulation although the number of genes observed in each category was slightly higher for M21, with the exception of “abiotic stress genes” where more up-regulated transcripts were observed for CC. Landrace 85-2 showed a poor response in the number of up-regulated transcripts in comparison to the other landraces. The greatest difference between the 3 landraces was observed for down-regulated transcripts on recovery irrigation. CC and 85-2 showed very similar patterns of down regulation where the numbers of transcripts in each category did not exceed 40. In contrast for M21 most categories showed down-regulation of between 40 to >70 transcripts representing a 2 fold difference in comparison to the other 2 landraces (Figure 6).

Genes associated with signal transduction such as calcium dependent protein kinases (CDPKs), G-proteins and receptor kinases were both up- and down-regulated under drought. M21 proved to be the landrace with most differentially expressed signaling genes at 17 days stress in comparison to the other two maize landraces. From 42 differentially expressed genes, seven were common to the two tolerant landraces and 27 genes were unique to M21 (Table S2) including genes encoding to: calcium binding protein, CDPK, calmodulin, GTP binding protein and phosphoinositides. For the recovery process, 170 differentially expressed genes related to signaling were identified, of which 32 were common to the two tolerant landraces including genes encoding receptor kinases, G-proteins, Ca^+2^ signaling, and phosphoinositides (Table S3).

In the heat shock category, a greater number of up-regulated genes encoding heat shock proteins under 17 days stress were observed, especially in the tolerant maize landraces (24 and 31 for CC and M21 respectively) in comparison to 85-2 (16). Perhaps surprisingly only HSP17 and LEA transcripts were up-regulated under stress and in common with HPS18, HSP70, DNaJ, HSF and dehydrins; these transcripts were strongly repressed on recovery. Other abiotic stress related genes, such as those for cold and drought/salt stress were in general induced under stress and repressed on recovery as would be expected. Several transport associated transcripts showed little change under drought but showed both up and down regulation on recovery irrigation including those associated with amino acids or metals, ABC, metabolite and Pi transporters. A pattern of up-regulation under stress and down-regulation on recovery was observed for the aquaporin genes. At 10 days stress, two up-regulated transcripts for tonoplast intrinsic proteins (TIPs) were identified only in the two tolerant landraces. One of these TIPs was also up-regulated at 17 days stress only in the two tolerant maize landraces. Two transcripts for nodulin-like intrinsic proteins (NIPs) were up-regulated at 17 days stress and at recovery irrigation were down-regulated. Interestingly the sugar transport associated genes were up and down regulated in both stages, reflecting the changes in sugar metabolism and transport which occur in relation to photosynthesis levels under drought and recovery. In relation to genes associated with detoxification, only peroxidases and thioredoxins genes were up-regulated. These genes showed both up and down regulation on recovery as did ascorbate and glutathione metabolism and dismutases and catalases genes. The only other transcripts which clearly showed repression under drought and induction on recovery were those associated with the peroxiredoxins (Table S3). Only one transcript for a peroxiredoxin was found to be induced at 10 days stress in M21 and this cultivar and CC showed the highest number of induced peroxiredoxin genes at recovery in relation to 85-2.

### Hormone related responses

Many hormone-related genes were found to be differentially expressed under stress and on recovery such as those related to abscisic acid (ABA), auxins, cytokinins and ethylene metabolism. The plant hormone ABA plays a central role in many aspects of response to various stress signals [15], [14] and has been shown to participate in drought and high salinity-tolerance mechanisms [31]. In this study, differential expression of genes involved in ABA metabolism was observed under drought stress and recovery irrigation. The 9-cis epoxycarotenoid dioxygenase (NCED) gene was up-regulated in 85-2 and M21 at 10 days stress. This enzyme is thought to be involved in the rate-limiting step in ABA biosynthetic pathway [32], [9]. At 17 days stress, a gene named ABA insensitive 3 (ABI3) was up-regulated in M21. This gene encodes a TF which may be involved in multiple hormonal signaling pathways related to ABA and auxins [33]. The *HVA22* gene known to be highly induced under drought, ABA, cold and salt stresses was also up-regulated only in M21. In addition at recovery irrigation, 11 down-regulated transcripts related to ABA metabolism were identified. During recovery irrigation, transcripts for AREB2 (ABA-responsive element binding protein), ABRE (ABA-responsive element binding protein) and a protein phosphatase 2C involved in ABA signal transduction were down-regulated in the three landraces and five transcripts for *HVA22* were down-regulated in at least one of the landraces.

Cytokinins are hormones that play an essential role in plant growth and development [34]. In this study, cytokinin related genes showed few changes under drought but were very strongly up-regulated on recovery with few genes down regulated. Under 10 days stress a gene for cytokinin oxidase was up-regulated only in 85-2. Cytokinin oxidase regulates the levels of cytokinin in plants by degrading it irreversibly [35]. At 17 days stress differentially expressed genes related to cytokinin signal transduction were identified. However, at recovery many up-regulated genes related to cytokinin signal transduction were observed: 14, 17 and 18 genes in 85-2, CC and M21 respectively (differences not statistically significant). Most were found to be response regulator genes (ARR). Only three genes related to cytokinin metabolism were down-regulated in at least one of the three landraces.

Auxin related genes showing induction of 2, 1 and 5 transcripts at 10 days stress in 85-2, CC and M21 respectively were also identified (differences not statistically significant). At 17 days stress 7 up regulated and 6 down regulated genes for auxin metabolism were identified and a transcript for GH3-like protein enzyme that conjugates amino acids to indole 3-acetic acid [36] was induced higher in M21. At recovery the number of differentially expressed genes increased 18 and 28 for up and down regulated genes respectively in at least one of the three landraces. Notably, the GH3 gene showed repression only in M21.

Ethylene is another hormone that is related to abiotic stress responses. However it was found that ethylene related genes showed no strong response under stress although a transcript for a putative Fe/ascorbate- dependent oxidoreductase was induced in CC and M21 at 10 days stress and at 17 days stress in M21. One ACC oxidase gene was repressed only in M21 whereas in CC it was found to be constitutive and in 85-2 the value was higher than 0.5 fold change. An ethylene response factor was also down-regulated in CC and M21 at 17 days stress. At recovery, many genes related to ethylene metabolism were found to be down regulated. The Fe/ascorbate- dependent oxidoreductase gene was repressed under this treatment in CC and M21, but not in 85-2. Transcripts of ACC oxidases were constitutively expressed in M21 but repressed in CC. A down-regulated transcript for the ethylene response factor (ERF) was identified in 85-2 and another in 85-2 and M21. However, a different ERF gene was also induced in CC and M21 at recovery.

### Transcription factors

Table 1 compares the number of differentially expressed transcription factor (TF) genes for each of the principal TF gene families and for each landrace. All TF gene families analyzed showed differential expression in the three landraces with differences in the patterns of induction/repression. In general the response of each landrace followed the pattern described previously for numbers of differentially expressed transcripts: 85-2 low, CC, intermediate and M21 high. A total of 121 differentially expressed genes encoding TF were identified in this study under drought stress. Among these, tolerant landraces (CC and M21) showed more genes induced and repressed for bHLH, WRKY, MYB, C2C2 and C2H2, HB and CCAAT (HAP2) TF families (24 and 78 respectively) compared to 85-2. Further, more members of the AP2/EREBP, HB and MADS families were up-regulated specifically in M21 under stress. Both CC and M21 had greater numbers of up- and down-regulated TFs in the unclassified group as compared to 85-2. On recovery irrigation, 202 differentially expressed genes encoding TF were identified. The tolerant landraces showed significantly different responses with more induced and repressed genes (CC 104 and M21 139 TFs respectively) compared to the susceptible landrace 85-2 for AP2, NAC, MADS, CCAAT, HB, bHLH, bZIP TF families which most of them were induced under stress but repressed on recovery irrigation. One CCAAT family member, HAP3 previously shown to confer drought tolerance by overexpression of NF-YB (HAP3) in *Arabidopsis* and maize [37] is specifically down-regulated on recovery in M21 whereas transcripts for HAP2 and HAP5 were up-regulated in CC and M21 also after recovery irrigation. The AP2/EREBP and MADS families also showed patterns specific to M21 on recovery irrigation. M21 showed the greatest number of responsive TF gene families, suggesting that this is the most responsive landrace at the level of differential gene expression under drought stress as well as under recovery irrigation.

**Table 1:** Principal transcription factor gene families differentially regulated under drought stress and recovery irrigation.

Treatment	Drought stress						Recovery irrigation					
Cultivar	85-2	85-2	CC	CC	M21	M21	85-2	85-2	CC	CC	M21	M21
Regulation	Up	Down	Up	Down	Up	Down	Up	Down	Up	Down	Up	Down
AP2/EREBP	1	0	2	0	4	1	1	2	3	1	5	6
bHLH	1	0	2	2	6	3	1	2	6	5	10	8
bZIP/Putative bZIP	2	0	0	0	2	1	3	1	5	2	5	6
C2C2	0	1	2	1	3	0	0	2	5	5	6	5
C2H2	0	2	0	3	0	1	1	5	7	5	7	5
CCAAT:	0	0	1	0	1	1	0	0	2	1	2	3
-HAP2	0	0	1	0	1	1	0	0	1	0	1	0
-HAP3	0	0	0	0	0	0	0	0	0	0	0	3
-HAP5	0	0	0	0	0	0	0	0	1	1	1	0
DNA binding	1	1	0	2	3	0	3	3	6	5	9	5
Finger TF	0	2	0	2	0	2	0	2	1	2	1	2
G2 like	1	3	1	4	1	2	1	4	3	5	3	3
HB	3	0	3	2	5	1	4	5	5	7	8	6
HD leucine zipper	1	0	1	0	1	0	1	0	1	1	1	1
MADS	3	0	2	0	6	0	3	4	2	4	6	7
MYB	3	3	2	4	7	5	5	6	7	8	11	12
NAC	0	0	1	1	0	0	1	2	1	3	2	3
Unclassified	2	2	5	3	6	5	7	7	18	11	24	16
WRKY	0	2	0	3	0	1	2	2	1	4	1	3
Zinc finger/HD	1	0	0	2	1	2	3	2	9	2	9	6
Total	19	16	22	29	46	24	36	49	83	71	111	98

## Discussion

The aim of this study was to compare changes at the physiological and global gene expression levels of two drought tolerant and one susceptible maize genotype in response to the gradual application of drought stress, in order to identify the general responses of maize to drought and possible differences in the mechanisms employed to achieve tolerance. Although some genetic variation exists within each landrace, the repetition of the drought experiment in 2 different years and the use of replicates of each landrace to obtain physiological and gene expression data produced consistent landrace specific data. Monitoring of soil water potentials throughout the application of drought stress and on recovery ensured that levels of stress were adequate and equivalent for all 3 landraces.

Physiological responses: Photosynthesis, stomatal conductance and water potential

It is well documented that upon water deficit, most plants respond rapidly by stomatal closure to avoid excessive water loss and by establishing physiological and molecular responses to prevent irreversible damage to the photosynthetic machinery (for a review see [9]). These two processes are closely linked since stomatal closure results in a decline in the rate of photosynthesis [38], [39]. Therefore, leaf water potential, stomatal conductance and rate of photosynthesis were monitored at different stages throughout the experiment and revealed different responses in the 3 landraces for these physiological parameters. M21 showed a more rapid and drastic reduction in stomatal conductance and rate of photosynthesis than CC and 85-2. CC showed a more gradual and less pronounced decline in leaf water potential, rate of photosynthesis and stomatal conductance at 10 days stress, whereas at 17 days stress the rate of photosynthesis and stomatal conductance dropped sharply. The susceptible landrace, 85-2 had the highest drop in leaf water potential which correlated with a lower decrease in stomatal conductance. In previous work the characteristic of drought resistance called “latency” observed in M21, was associated with early stomatal closure in comparison to the susceptible controls and that stomatal hypersensitivity was a trait common to several drought resistant maize lines [40]. The M21 strategy to sharply drop photosynthesis rate may be advantageous when short periods of severe drought stress are experienced, however it could have a negative effect under prolonged drought stress even though the overall stress is less severe. Under the latter conditions the CC strategy of gradual reduction in photosynthesis rate and higher leaf water potential may allow the plant to survive for longer periods of low water availability. At recovery irrigation, both CC and M21 show a rapid and strong increase in photosynthesis as compared to the response in 85-2, suggesting that the drought-tolerant genotypes may share a mechanism of rapid recovery after drought not present in the susceptible landrace.

### Molecular drought stress responses common to the three maize genotypes

Analysis of the general overview of the global changes in gene expression in response to drought for the tolerant and susceptible landraces shows that there are several common alterations, albeit to a significantly different degree in terms of the number of transcripts differentially expressed and in the expression levels of genes involved in different metabolic and cellular processes (Figure 4). The earliest response to water deficit is stomatal closure to protect the plants from extensive water loss [9], [14] and consequently the inhibition of photosynthesis [9]. In this respect, the first obvious common response of the three genotypes is the decrease in transcript level of photosynthesis-associated genes during drought stress. In particular, genes encoding components of photosystem II, and to a lesser extent of photosystem I, are repressed during drought stress. A reduction in the components of photosystems I and II would prevent the photo-oxidation of the photosynthetic apparatus and the formation of free radicals that are harmful for the cell, although as discussed below there were significant differences in the expression of photosynthesis-associated genes that were observed only in the drought tolerant genotypes or specific to either of them. Another feature in the general maize gene response to drought was the induction of genes encoding HSP and LEA proteins. Genes encoding HSP17, HSP22, HSP70 and HSP90 were induced in the three genotypes under drought stress. These proteins prevent detrimental effects of stress by preventing protein aggregation, protecting non-native enzymes from degradation and assisting in protein refolding [41]. Induction of these genes under drought stress was observed in previous studies on drought stress in barley [21] and rice [19] dehydration in *Arabidopsis* [16] and PEG treatment in maize [42]. Interestingly, only two LEA genes, one belonging to group 1 and the other to group 3, were identified as induced in all three genotypes, suggesting that as a whole the LEA protein family might not play a major role, at least under our experimental conditions, in the general drought stress response in maize.

Plant growth and response to stress conditions is largely under the control of hormones [14]. In particular, ABA has been associated with the promotion of stomatal closure and plays an important role in the tolerance response of plants to drought and high salinity [43]. In the present study, genes encoding enzymes related to ABA synthesis (ZEP and NCED) were induced at 10 days stress in the three landraces. These genes were shown to be up-regulated by dehydration in *Arabidopsis* [43]. As mentioned above, NCED is a key enzyme of ABA biosynthesis; *At-NCED3* was strongly induced by dehydration and high salinity and its overexpression improved dehydration stress tolerance in transgenic plants, indicating the important role in ABA accumulation during dehydration [43]. The induction of *HVA22* under environmental stresses such as ABA, cold and drought has been reported in barley [44]. It was also reported that the ectopic expression of *ABI3* conferred a freezing tolerance in transgenic ABI3 *Arabidopsis* plants [45]. The up-regulation of transcripts for ABI3 and *HVA22* exclusively in M21 under stress suggests the existence of specific ABA signaling stress response in this landrace that could be important for the drought tolerance.

Reduction in carbon fixation and the inhibition of photosynthetic activity by drought also alters the carbohydrate metabolic equilibrium [46]. For plants, carbohydrate based regulation represents an especially valuable mechanism for adjusting to environmental changes [47]. An increase in β-amylase transcripts in all 3 landraces under drought suggests that when levels of photosynthesis drop, carbohydrates stored as starch may be mobilized from the chloroplasts. This could lead to the increase in glucose levels observed for M21 and 85-2.

Glucose in addition to a structural role, functions as a signal molecule in both hexokinase dependant and independent pathways [48]. Although hexokinase transcripts were up-regulated under drought stress the genes encoding the other enzymes needed to produce MI from glucose were down regulated suggesting that the increase in MI content observed at 10 days in CC and 85-2 and at 10 and 17 days in M21 could be the result of changes regulated at the translational or post-translational level. The fact that M21 has a high MI content could indicate another drought tolerance strategy, since MI is implicated in many aspects of metabolism including: osmoregulation, auxin physiology, cell wall and membrane metabolism and signaling among others [49]. Increases in transcript levels in response to drought, both at the transcriptional and/or posttranscriptional level, requires the participation of components of signaling pathways that activate transcription and/or mRNA stabilization. In this study components of signal transduction pathways related to Ca^+2^ signaling and G-proteins (CDPK, Ca^+^2-binding EF, Rho and Rab GDP dissociation inhibitor and calmodulin) were the only ones identified as differentially regulated common in the three genotypes under stress. The involvement of Ca^+2^ signaling in response to osmotic and ionic stress has been well documented [15]. Signal transduction networks usually include TFs and their cognate cis-acting elements [43] that activate a cascade of genes encoding proteins and enzymes that may act together to enhance tolerance to multiple stresses [50]. Previous studies have revealed that plant responses are complex requiring the participation of several TFs, some of which are transcriptionally activated during drought stress. Most of these TFs fall into several large families, such as AP2/ERF, bZIP, NAC, MYB, CysHis2 zinc finger and WRKY gene families [51]. The three maize genotypes analyzed in this study showed a common up-regulation of several genes encoding TF belonging to the C2H2, G2-like, HB, MADS and MYB gene families and a homeodomain leucine zipper protein Hox7, which could be considered to be induced in the general response of maize to drought. Some of the genes encoding TFs common to the three maize landraces, such as MADS and homeodomain leucine zipper TFs, were also up-regulated in wheat [22], MYB in *Arabidopsis* [16], and C2H2 under PEG stress in maize [42]. This suggests that some of the responses in differential gene expression to drought stress are probably modulated by the same types of TFs and involve similar signal transduction pathways.

### Differential responses among the three landraces under drought stress

**Figure 7:**
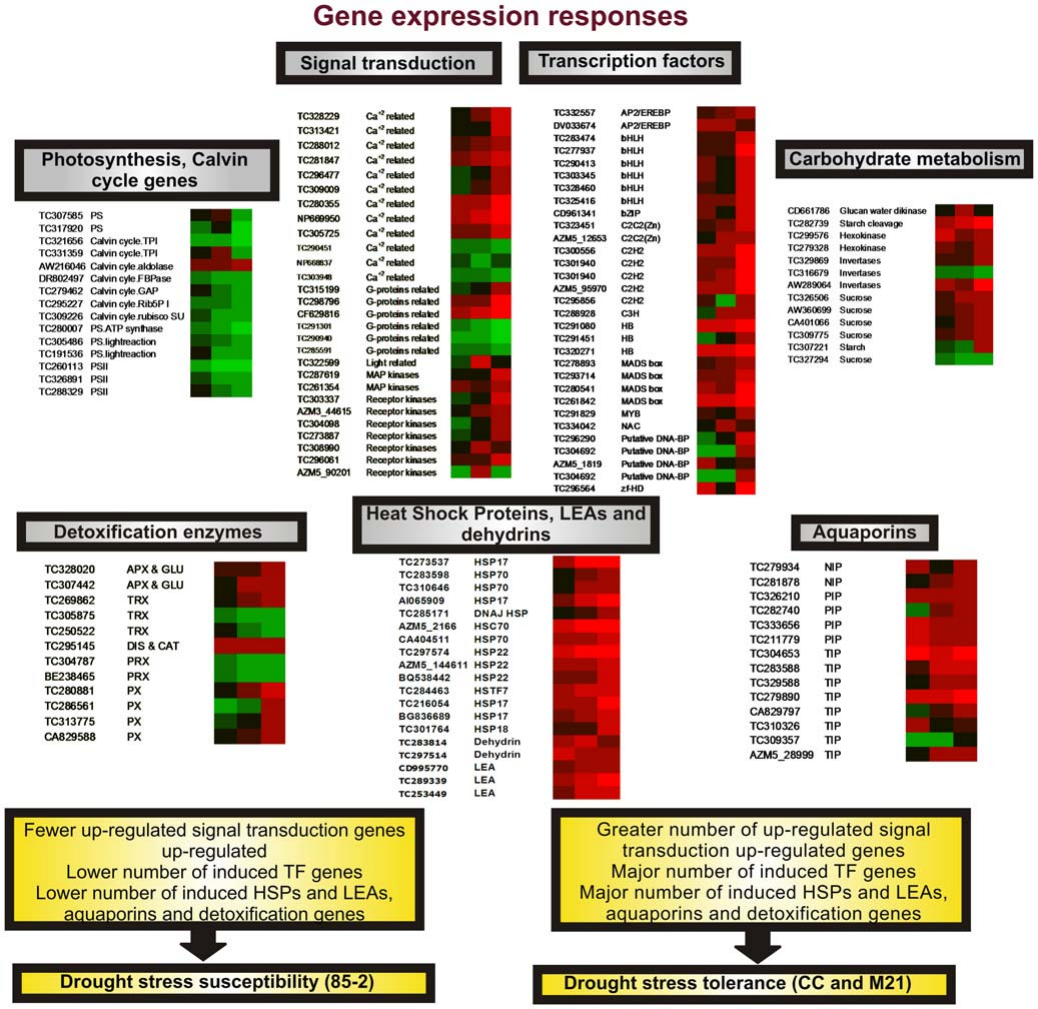
General view of gene expression responses in the three maize landraces under drought stress. Gene expression was monitored at 10 and 17 days stress and differences in gene expression levels were observed between the landraces. Transcripts encoding signal transduction, transcription factors, HSP, detoxification enzymes and aquaporins are shown. Gene identifiers correspond to the accession numbers from the corresponding databases as reported in Maize Oligonucleotide Array Annotation GAL files version 1.13 (http://www.maizearray.org/maize_annotation.shtml). Microarray data were visualized using the FiRe 2.2 Excel macro [67]. A ≥2 fold change is shown in red, a fold change ≤0.5 in green and no change in black (FDR ≤0.05). Left column: 85-2, middle column: CC and the right column: M21. PS: photosystem, TPI: triosephosphate isomerase, FBPase: fructose-1,6-bisphosphatase, GAP: glyceraldehyde 3-phosphate dehydrogenase, Rib5PI: ribose 5-phosphate isomerase-related, Rubisco SU: ribulose bisphosphate carboxylase small subunit, PSII: photosystem II, MAP kinase: mitogen-activated protein kinase, AP2/EREBP: AP2/Ethylene-responsive element binding protein family, bHLH: Basic Helix-Loop-Helix family, C2C2: C2 domain-containing protein, similar to zinc finger and C2 domain protein, C2H2: C2 domain-containing protein, similar to zinc finger and C2 domain protein, C3H: zinc finger (CCCH-type) family protein, HB: Homeobox transcription factor, Putative DNA BP: putative DNA binding protein, zf-HD: zinc finger homeobox, APX & GLU: ascorbate peroxidase and glutathione related, TRX: thioredoxin, PRX: peroxiredoxin, PX: peroxidase, HSP17: 17 kDa class I heat shock protein, HSP70:heat shock protein 70, HSC70: heat shock cognate 70 kDa protein, HSP22: 22.0 kDa ER small heat shock protein, DNA J HSP: DNAJ heat shock protein, HSP18: 18.1 kDa class I heat shock protein, LEA: late embryogenesis abundant protein, NIP: NOD26-like membrane integral protein, PIP: Plasma membrane intrinsic protein, TIP: tonoplast intrinsic protein.

In order to elucidate differences between the tolerant and the susceptible landraces we identified gene families that are over-represented or have differences in their expression level in the tolerant genotypes with respect to the susceptible one (Figure 7). In fact, one might expect more changes in the tolerant cultivars, which should carry alleles of genes that contribute to increased tolerance [52]. Although it is quite possible that some genes responsible for drought tolerance might not be inducible or repressible during stress, the identification of differentially expressed genes in drought tolerant genotypes, could provide important information about the metabolic and cellular processes that are ultimately responsible for stress tolerance. In this respect, TFs are important in regulating the expression of downstream stress-regulated genes [53]. It was found eighteen TF genes are differentially expressed under drought stress in both tolerant genotypes but not in 85-2 such as some members of the AP2, bHLH, C2C2, C2H2, C3H, zinc finger, CCAAT binding factor (HAP2) and WRKY gene families (Table S1 and Table S2). Of these, one AP2/EREPB, one C2C2 (H-protein promoter binding factor 2b), one C2 domain containing C2H2 zinc finger family, one zinc finger CCCH-type and the CCAAT binding protein NF-YA were induced under stress only in the two tolerant genotypes. C2C2 and CCAAT families have been proposed to play an important role in drought tolerance [54]. It was also reported that the expression of *NFYA5*, a member of NF-YA (HAP2) was strongly induced by drought stress or ABA treatments in *Arabidopsis* and suggested to play an important role in controlling stomatal aperture and drought resistance [53]. On the other hand, AP2/EREPB domain proteins include DREB or CBF proteins which bind to dehydration response elements (DRE) or C-repeats [15], also shown to be involved in improved stress tolerance to drought, high salinity and freeze in *Arabidopsis* [43] and rice [55]. Additionally, 37 and 7 TF genes were specifically up-regulated under stress for M21 and CC respectively. In M21 some of these genes showed a 3- to 8-fold higher change in expression to 85-2 and CC (Table S1 and Table S2). These TFs could be important for the regulation of drought stress responsive genes and might be involved in the tolerance mechanism. The higher number of up-regulated TF genes observed for M21 could explain the more rapid and wide-ranging responses observed in this landrace and therefore its tolerance mechanism.

In CC a remarkable difference was the induction of a NAC TF gene. NAC proteins are known to function as transcription activators in cooperation with the ZFHD (zinc finger homeodomain) proteins and the overexpression of these genes significantly increased drought tolerance [43]. Different alleles of the same TF genes may also produce different responses between the tolerant landraces and between the tolerant and susceptible landraces as well as a careful orchestration of gene expression leading to tolerance or susceptibility to a greater or lesser extent. Taken together these results suggest that some tolerance mechanisms are similar whereas others are specific to each landrace.

Drought stress tolerance requires changes in water transport to allow cells and tissues to adapt to the stress situation. Aquaporins facilitate osmosis by forming water-specific pores as an alternative to water diffusion through the lipid bilayer thus increasing the permeability of the membrane [15]. Although several aquaporin genes were differentially expressed in the three genotypes, CC and particularly M21 had more up-regulated genes encoding NIP, TIP and PIP aquaporin as compared to 85-2. These results suggest that in the tolerant genotypes the activation of greater number of aquaporin genes would facilitate water flux to maintain cellular homeostasis.

A secondary effect of dehydration is the increase in reactive oxygen species (ROS) [15] and consequently the requirement of an enhanced activity of antioxidant enzymes [9] to protect cells from oxidative injury under drought stress [50]. In this case, increased transcript levels for thioredoxin and peroxidases were observed for both tolerant genotypes but mainly in M21, whereas in the susceptible line the up-regulation of these genes was rare. These results suggest that the tolerant genotypes and in particular M21 activate the expression of genes that could allow them to better cope with ROS.

Although it was observed that the induction of HSPs is a common response in maize, a greater number of genes encoding HSPs were induced under drought stress in the tolerant landraces (24 and 31 for CC and M21) as compared to the susceptible one. In particular, a higher number of members of the HSP17 family were induced in the tolerant landraces. A positive correlation between the levels of several HSPs and stress tolerance has been previously reported [56], [1], [15]. Since small HSPs have a long half life, it has been proposed that they might play an important role during stress recovery [56] therefore, signaling pathways that lead to an increased expression of small HSP might be preferentially activated in drought tolerant maize genotypes in comparison to susceptible ones.

Decrease photosynthetic activity under drought is due to reductions in stomatal conductance and Rubisco activities leading to lower carbon fixation [46] that consequently results in the over-reduction of components within the electron transport chain, generating ROS [14]. In our study, it was found that genes related to PSI and PSII were down-regulated mainly in the tolerant landraces, suggesting that these genotypes reduce the activity of the components of the PSs to avoid the generation of large amounts of ROS under drought stress. A similar situation was also observed in rice drought tolerant cultivars under drought stress, where a higher number of members of gene families related to PSI and PSII were down-regulated in tolerant rather than sensitive cultivars [52]. We also found that a larger number of Calvin cycle related genes such as transcripts for Rubisco, phosphoglycerate kinase, GADPH, TPI and FBPase were repressed under stress in both tolerant genotypes, and to a greater extent in M21, suggesting that a general repression of photosynthesis related genes occurs in maize under drought. Ten photosynthesis-related genes that were repressed at 17 days stress were induced at recovery most of them were shared between the two tolerant landraces. Together these results suggest that the maize drought-tolerant genotypes analyzed in this study (more evident in M21) more broadly reduce electron transport in the PSI and PSII systems, probably to reduce the effect of photoxidation and the synthesis of enzymes involved in carbon fixation to avoid spending energy and resources under conditions of low CO_2_ availability.

### Osmotic adjustment by proline accumulation in the three maize landraces under stress and at recovery

Proline is probably the most widely distributed osmolyte in plants [15] and is implicated in responses to various environmental stresses [14]. Besides osmotic adjustment other roles such as protection of plasma membrane integrity, as an energy sink or reducing power, a source for carbon and nitrogen, or a hydroxyl radical scavenger [15] as well as preservation of enzyme structure and activity [9] have been proposed for this molecule in osmotically stressed plant tissues. It was found that M21 had the highest accumulation of proline under 10 and 17 days stress, followed by CC, whereas 85-2 showed the lowest level of proline accumulation. The up-regulation of genes encoding P5CS in CC and a putative ornithine aminotransferase, involved in an alternative proline biosynthetic pathway (via ornithine aminotransferase) in M21 at 17 days stress could explain the higher accumulation of proline in the tolerant genotypes. P5CS is a rate-limiting enzyme for the biosynthetic pathway via glutamate in higher plants [57], [58] and it has been reported that concomitant to the accumulation of proline, an increase in the expression of P5CS is observed in a salt-tolerant genotypes but not in sensitive genotypes exposed to salt stress [57]. Our results suggest that both proline biosynthetic pathways are active in maize and that depending upon the genotype only one of them is activated during drought stress.

At recovery irrigation, a strong correlation between the decrease in proline content and the up-regulation of genes involved in proline degradation: pyrroline-5-carboxylate dehydrogenase (P5CDH) and the down-regulation of P5CS were observed in the three maize landraces. Although the proline content at recovery irrigation decreased in the three landraces, M21 had the highest content of proline after recovery irrigation. High proline content has been associated with increased recovery capacity [59].

### Differential molecular responses under recovery in the three maize landraces

**Figure 8:**
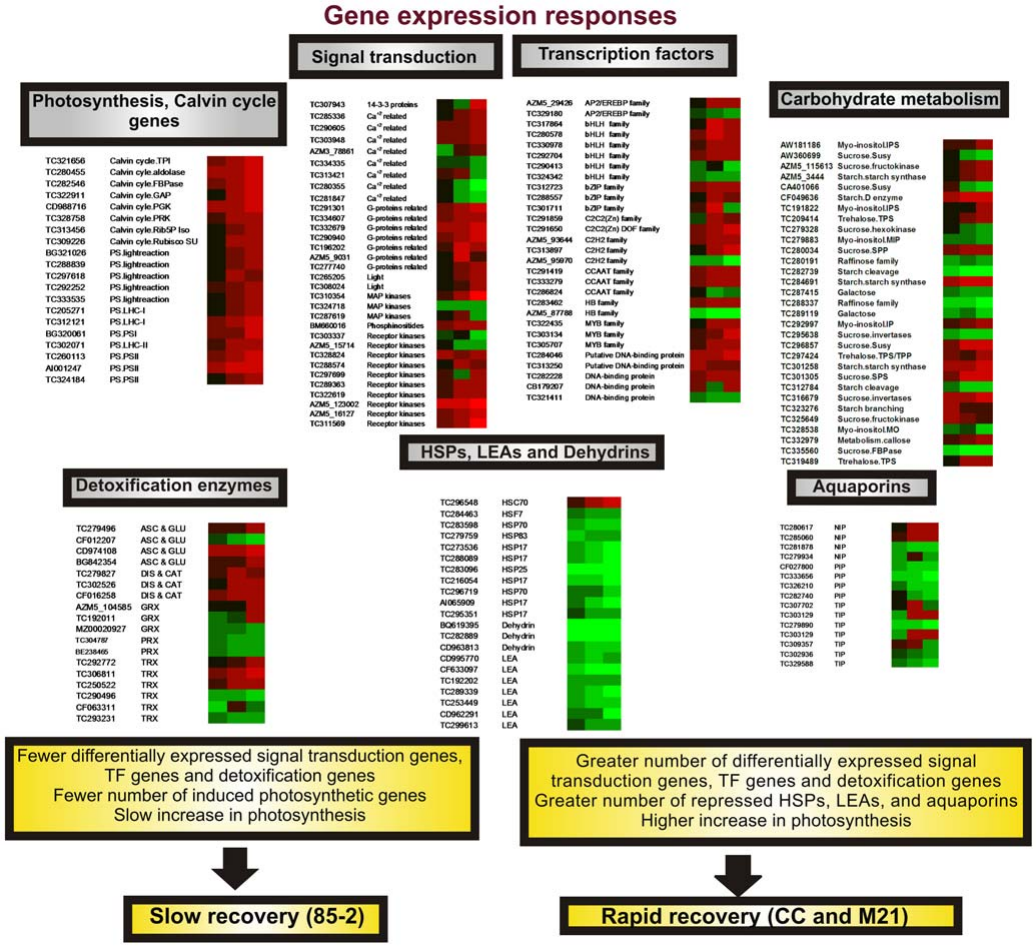
General view of gene expression in the three maize landraces on recovery. Gene expression was monitored at 10 and 17 days stress and differences in gene expression levels were observed between the landraces. Transcripts encoding signal transduction, transcription factors, HSP, detoxification enzymes and aquaporins are shown. Gene identifiers correspond to the accession numbers from the corresponding databases as reported in Maize Oligonucleotide Array Annotation GAL files version 1.13 (http://www.maizearray.org/maize_annotation.shtml). Microarray data were visualized using the FiRe 2.2 Excel macro [67]. A ≥2 fold change is shown in red, a fold change ≤0.5 in green and no change in black (FDR ≤0.05). Left column:85-2, middle column: CC and the right column: M21. TPI: triosephosphate isomerase, FBPase: fructose-1,6-bisphosphatase, GAP: glyceraldehyde 3-phosphate dehydrogenase, PGK: phosphoglycerate kinase, PRK: phosphoribulokinase, Rib5P Iso: ribose 5-phosphate isomerase-related, Rubisco SU: ribulose bisphosphate carboxylase small subunit, LHC-I: Light harvesting chlorophyll a/b binding protein of PSI, PSI: photosystem I, LHC-II: Light harvesting chlorophyll a/b binding protein of PSII, PSII: photosystem II, MAP kinase: mitogen-activated protein kinase, AP2/EREBP: AP2/Ethylene-responsive element binding protein family, bHLH: Basic Helix-Loop-Helix family, C2C2: C2 domain-containing protein, similar to zinc finger and C2 domain protein, C2H2: C2 domain-containing protein, similar to zinc finger and C2 domain protein, CCAAT: CCAAT-binding transcription factor, HB: Homeobox transcription factor, MIP:myo-inositol-1-phosphate synthase, Susy: sucrose-phosphate synthase, TPS: trehalose-6-phosphate synthase, SPP: sucrose-phosphatase, IP: nositol monophosphatase, TPS/TPP: trehalose-6-phosphate synthase/phosphatase, MO: myo-inositol monophosphatase, TPS: trehalose-6-phosphate synthase, ASC & GLU: ascorbate and glutathione related, GRX: glutaredoxins, PRX: peroxiredoxin, TRX: thioredoxin, HSC70: heat shock cognate 70 kDa protein, HSF7: heat shock factor protein 7, HSP70:heat shock protein 70, HSP83: heat shock protein 83, HSP17: 17 kDa class I heat shock protein, HSP25: 25.3 kDa small heat shock protein, LEA: late embryogenesis abundant protein, NIP: NOD26-like membrane integral protein, PIP: Plasma membrane intrinsic protein, TIP: tonoplast intrinsic protein.

On recovery a total of 2567 and 2765 up- and down-regulated transcripts were identified in the three genotypes, of which 1466 genes were found to be inversely regulated between stress and recovery (induced during stress and repressed during recovery and viceversa). Figure 8 shows a general scheme of the responses observed in the three landraces during the recovery process. The observation that the greatest number of differentially expressed genes was found at recovery, suggests a rapid and global re-activation of general plant metabolism following severe stress. As expected, the number of down-regulated genes encoding HSPs was greater in the tolerant than in the susceptible landraces, particularly in M21. Further, the two tolerant landraces shared differentially expressed genes related to signaling including receptor kinases, G-proteins, Ca^+2^ signaling, and phosphoinositide metabolism during recovery irrigation. The fact that CC and M21 have a higher number of induced signaling genes at recovery (48 and 47 genes respectively) than 85-2 (32 genes) indicates the possibility that the tolerant genotypes adjust their metabolism more efficiently during the recovery process. Genes related to carbon metabolism were up-regulated in tolerant landraces, however in 85-2 most of these genes were constitutive and this could be one of the key differences between tolerant and susceptible responses. Genes encoding Calvin cycle enzymes, PSI, PSII and photosynthesis related enzymes were also up-regulated mainly in the tolerant genotypes on recovery. The up-regulation of genes involved in photosynthesis and Calvin cycle is in accordance with the increase in the rate of photosynthesis and stomatal conductance observed after recovery in CC and M21. In this context, the up-regulation of peroxiredoxins genes shown to protect DNA, membranes and certain enzymes against damage by removing H_2_O_2_ and hydroxyl radicals [15], during recovery irrigation in the two tolerant landraces, could represent a protective mechanisms against the production of ROS during a rapid re-activation of photosynthetic activity. The down-regulation during recovery of genes encoding aquaporins, supports their importance in stress tolerance. The expression patterns of genes encoding TFs were distinct for the three landraces, suggesting different responses during recovery. A greater number of TF genes including bHLH, MADS and MYB were found to be both up- and down regulated in the tolerant landraces in comparison to 85-2.

A significant difference was found in the recovery response of M21 which showed the greatest changes in gene expression in almost all of the functional categories of the three genotypes (except for signaling), suggesting that this genotype possess a recovery mechanism that responds rapidly by activating metabolic processes on recovery. Expression changes in CC also reflect these responses albeit at a lower level and 85-2 is the least responsive even though it shares some common genetic background with M21.

### Conclusions

Differences in rates of photosynthesis, stomatal conductance, sugar and proline accumulation and gene expression patterns were identified between the 3 landraces. In many cases, in comparison to the tolerant landraces, 85-2 failed to respond or responded more weakly. Important differences were also noted between the tolerant landraces that probably underlie different mechanisms of achieving tolerance: CC may have an advantage under prolonged drought periods due to a gradual reduction of photosynthesis and stomatal conductance, whereas M21 with the capacity for latency, with a rapid reduction of photosynthesis and efficient recovery responses may perform better under short periods of severe drought stress. Although necessarily the most outstanding differences have been emphasized in this study, subtle differences between the landraces should not be overlooked. Differences in response mechanisms were also supported by the detailed changes in gene expression patterns under drought conditions. Modulation of a greater number of differentially expressed genes from different TF gene families could be an important characteristic of the tolerant landraces, many belonging to families previously implicated in stress responses such as members of the AP2/EREBP, bHLH, HB, CCAAT and MYB TF gene families. Furthermore, the genes encoding hormones, aquaporins, HSPs, LEAs and detoxification enzymes were induced to a greater extent in the tolerant landraces again suggesting more efficient responses in these genotypes. In the case of recovery from the drought stress, the most important feature was the speed and scope of changes in gene expression, which differed between the 3 genotypes. This report emphasizes the most outstanding differences between drought-tolerant and susceptible genotypes and identified potential regulators of the drought and recovery processes in maize. The task in hand is now to characterize the expression patterns and responses unique to each landrace and the genes or specific alleles involved in order to compare with other commercial maize germplasm and suggest possible breeding or transgenic strategies to improve drought tolerance. Modulation of expression of specific transcription factor genes has already proved successful in improving drought stress [41]. In this report several additional TF families were identified and the regulatory effects of these genes in particular should be studied in more detail.

## Materials and Methods

### Plant material

Three Mexican maize genotypes were used for this work. Cajete criollo (CC) and Michoacán 21 (M21) are considered to be drought tolerant landraces and were supplied by the International Maize and Wheat Improvement Center (CIMMYT), whereas 85-2 is considered to be susceptible from field observations, and was supplied by the Instituto Nacional de Investigaciones Forestales, Agricolas y Pecuarias (INIFAP)-Mexico.

### Growth conditions

Seeds were treated with NaOCl (10%) for 30 min then washed with distilled water before sowing. Plants were grown in 15 L plastic pots in a substrate of 92.46% sand and 7.44% clay under greenhouse conditions in the months of July to September in 2005 and 2006 at CINVESTAV, Irapuato, Mexico. Temperatures were between 19°C to 32°C and relative humidity was 60±5%. Maize plants were watered daily to soil capacity until application of stress, and fertilization was applied using a slow release fertilizer (Triple 17, Profer Mix, 4 g for each pot-17:17:17 NPK). Long Ashton Solution [60] was added once a week until application of drought stress.

### Drought stress treatments

Thirty day old plants were subjected to a progressive water deficit by leaving them unwatered for 17 days (severe stress) and then given recovery irrigation. Control plants were watered daily to maintain soil water content close to field capacity. Soil and leaf water potentials were measured daily for the 17 days of the drought treatment. At day 0, 10 and day 17 of drought stress and following the recovery irrigation samples for RNA extraction were collected.

### Leaf (ψ_l_) and soil (ψ_s_) water potential and gas exchange

Leaf water potential (ψ_l_) was measured both pre-dawn (6 am) and at midday (12 pm) in control and stressed plants with a psychrometer Model C-52 sample chamber (WESCOR, Inc., Logan, Utah) and a dew point microvoltimeter (model HR-33T, WESCOR, Inc., Logan Utah), for each individual plant on the most recent fully expanded leaf. Soil water potential (ψ_s_) was measured using Model PST-55 psychrometers (WESCOR, Inc., Logan, Utah) placed in the center of each pot at a depth of 15.5 cm.

Photosynthesis and stomatal conductance were analyzed using a portable Li-6200 photosynthesis system (Li-Cor, Lincoln, NE, USA) every 2 hours between 7 am to 7 pm on the most fully expanded leaf for both control and drought stressed plants at 0, 10 and 17 days drought stress and on recovery irrigation. The values were normalized with a foliar area of 9 cm^2^.

### Microarray design

The Maize Oligonucleotide Array (MOA) from http://www.maizearray.org was used in this study. The MOA contains about 57000 individual spots on two slides (A and B) and putatively contains all maize genes identified when slides were obtained. Array annotation and composition is available at www.maizearray.org. A loop design was used in order to contrast the gene expression differences between genotypes under each treatment. Single samples analyzed included two independent biological replicates and two technical replicates. The biological replicates were obtained by pooling the leaves of five representative plants from each cultivar under a particular treatment (0, 10 and 17 days of drought stress and recovery irrigation). A total of twenty-four sets (48 slides) of microarray hybridizations were carried out, including direct and dye swap comparisons.

### RNA isolation and labeling, microarray hybridization and image processing

Total RNA from pooled leaves of 5 control and 5 stressed plants for each cultivar and each time point was isolated using the TRIZOL reagent (Invitrogen) and then re-purified with the Concert Plant RNA Purification reagent (Invitrogen). To ensure the purification of high quality RNA samples, the RNeasy MinElute Cleanup kit (Qiagen) was used following the manufacturer's instructions. Purified total RNA was then labeled according to the protocols recommended at http://www.maizearray.org. Probe concentrations were determined in a NanoDrop spectrophotometer ND-100 (NanoDrop Technologies Inc., Wilmington-DE, USA). Three micrograms of cRNA of each probe was used per slide. Hybridization, washing and scanning were performed as described in [29].

### Microarray normalization and data analysis

Raw data from the 48 slides was imported into the R 2.2.1 software (http://www.R-project.org) and background correction was carried out. Normalization of the corrected signal intensities within slides was carried out using the “printtiploess” method [61] and between slides using the Aquantile method. Both methods were implemented using the LIMMA package [62]. All microarray data reported in this manuscript is described in accordance with MIAME guidelines and have been deposited in NCBI's Gene Expression Omnibus.

The analysis was basically performed as described in [29]. A mixed linear model analysis [63], [64] was conducted for each printed oligonucleotide by using the SAS mixed procedure (SAS 9.0 software, SAS Institute Inc., Cary, NC, USA). Direct comparisons between genotypes under a particular treatment were done on each slide. The design permitted the evaluation of the differences in gene expression between the three genotypes under a specific drought stress or recovery irrigation treatment but also whether differences were treatment dependent by including the data from different treatments in the mixed model and looking for gene specific effects. Normalized data were log2 transformed and then fitted into mixed model ANOVAs using the Mixed procedure with two sequenced linear models considering as fixed effects the dye, cultivar, treatment and cultivar*treatment. Array and array*dye were considered as random effects. The Type 3 F-tests and p-values of the genotype*treatment and genotype model terms were explored and significance levels for those terms were adjusted for by the False Discovery Rate (FDR) method [65]. Estimates of differences in expression were calculated using the mixed model. Based on these statistical analyses, the spots with an FDR less or equal to 5% (FDR≤0.05) and with changes in signal intensity between stressed and control leaves of 2 fold or higher were considered as differentially expressed.

### Accession Numbers

The microarray data have been deposited in NCBI's Gene Expression Omnibus [66] and are accessible through GEO Series accession number: GSE14728 (http://www.ncbi.nlm.nih.gov/geo/query/acc.cgi?acc=GSE14728).

### Functional annotation and metabolic pathway analysis using MapMan software and BioMaps

Functional annotation and metabolic pathway analysis were performed as described by [29]. Genes differentially expressed according to the selected parameters (FDR <0.05 and Fold ±2) were visualized and clustered with the standard correlation method using GeneSpring 7.0 software (Silicon Genetics, Redwood City, CA). FiRe 2.2 macro Excel^®^ (Microsoft) [67] was used to facilitate the handling of the microarray information. Due to the limited functional annotation in maize, the functional classification in the mapping files that structure the Arabidopsis genes from the Affymetrix ATH1 array into distinct metabolic and cellular processes from the MapMan program [30] was used. Differentially expressed maize genes were functionally annotated by performing a BLAST alignment against the TAIR Arabidopsis database release 6.0 (www.arabidopsis.org) and to PLANTA database (TIGR).

The annotations of the mapping files for the best match to the TAIR protein database (with at least an Expected Value of 1.0E-10) were assigned to the corresponding maize ortholog. MapMan software [30] was employed to show the differences in gene expression in different cellular and metabolic processes. Ratios were expressed in a log2 scale for importing into the software and changes in expression were displayed via a false color code [30].

In addition to the MapMan software, the microarray data was analyzed using a tool called BioMaps [68] at the Virtual-Plant site (www.virtual plant.org). This tool helps relate differential expression data with functional categories based on the functional classification by the Munich Information Center for Protein Sequences (MIPS) annotation, taking into account the best match to the TAIR protein database and was utilized to identify the common functional categories related to drought stress among the three landraces and/or the tolerant landraces.

### Application of Pearson's Chi- squared test

In order to verify the statistically significant difference among the three landraces of the differentially expressed genes along the microarray analysis a Pearson's Chi- squared test was performed using R version 2.7.1 (2008-06-23) software (http://www.R-project.org) for some functional category and for each treatment of drought stress and recovery irrigation.

## Supporting Information

Figure S1 Validation of the microarray analysis by qRT-PCR. (*) Genes that have no significant values in the microarray (FDR≥0.05). TC279430: Nitrate reductase, CD952060: putative ERD4 protein, AZM5_14615: Ribulose bisphosphate carboxylase/oxygenase activase, CF628075_root: cold-regulated protein, TC260113: probable photosystem II oxygen-evolving complex protein 2 precursor, TC191404: Chlorophyll a-b binding protein 48, chloroplast precursor, TC312355: ferredoxin, AZM5_46378: putative receptor ser/thr protein, NP161441|AF099387.1|AAF04662.1: R2R3MYB-domain protein, TC318956: proline transport protein-like, DR802497: fructose-1, 6-bisphosphatase, TC310514: putative calcium-dependent protein kinase, TC296224: Putative leucine-rich repeat transmembrane protein kinase, TC321656: putative Triosephosphate isomerase, TC191823: myo-inositol 1-phosphate synthase, TC283096: heat shock protein 26. (CC:Cajete criollo, M21: Michoacán 21).
(5.08 MB TIF)

Table S1 Transcript levels of significant genes in the maize oligonucleotide microarray at 10 days stress.
(0.90 MB XLS)

Table S2 Transcript levels of significant genes in the maize oligonucleotide microarray at 17 days stress.
(1.39 MB XLS)

Table S3 Transcript levels of significant genes in the maize oligonucleotide microarray at recovery irrigation.
(2.68 MB XLS)

Table S4 BioMaps analysis of the common up-regulated genes among the three maize landraces at 17 days stress
(0.04 MB DOC)

Table S5 BioMaps analysis of the common down-regulated genes among the three maize landraces at 17 days stress
(0.03 MB DOC)

Table S6 BioMaps analysis of the up-regulated genes common in the tolerant landraces at 17 days stress.
(0.04 MB DOC)

Table S7 BioMaps analysis of the down-regulated genes common in the tolerant landraces at 17 days stress.
(0.04 MB DOC)

Table S8 BioMaps analysis of the common up-regulated genes among the three maize landraces at recovery irrigation.
(0.03 MB DOC)

Table S9 BioMaps analysis of the common down-regulated genes among the three maize landraces at recovery irrigation.
(0.04 MB DOC)

Table S10 BioMaps analysis of the up-regulated genes common in the tolerant landraces at recovery irrigation.
(0.06 MB DOC)

Table S11 BioMaps analysis of the down-regulated genes common in the tolerant landraces at recovery irrigation.
(0.05 MB DOC)

Table S12 Chi-square test.
(0.03 MB XLS)
