# Supplementary figures and images for: Analysis of Gene Expression and Physiological Responses in Three Mexican Maize Landraces under Drought Stress and Recovery Irrigation
Source: PLoS One. 2009 Oct 30;4(10):e7531. doi: 10.1371/journal.pone.0007531 (PMC2766256; doi:10.1371/journal.pone.0007531)

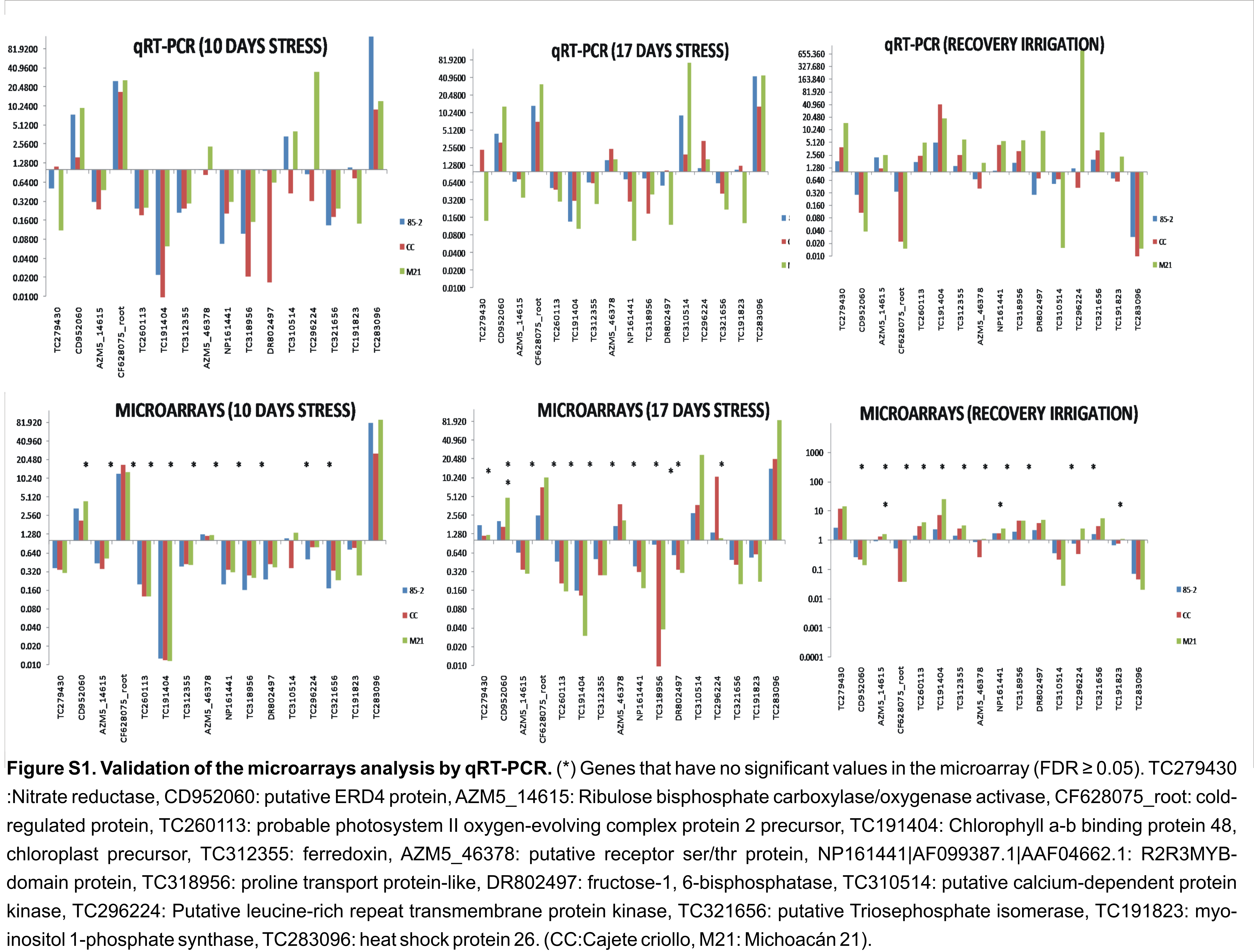

Supplement: Figure S1 — Validation of the microarray analysis by qRT-PCR. (*) Genes that have no significant values in the microarray (FDR≥0.05). TC279430: Nitrate reductase, CD952060: putative ERD4 protein, AZM5_14615: Ribulose bisphosphate carboxylase/oxygenase activase, CF628075_root: cold-regulated protein, TC260113: probable photosystem II oxygen-evolving complex protein 2 precursor, TC191404: Chlorophyll a-b binding protein 48, chloroplast precursor, TC312355: ferredoxin, AZM5_46378: putative receptor ser/thr protein, NP161441|AF099387.1|AAF04662.1: R2R3MYB-domain protein, TC318956: proline transport protein-like, DR802497: fructose-1, 6-bisphosphatase, TC310514: putative calcium-dependent protein kinase, TC296224: Putative leucine-rich repeat transmembrane protein kinase, TC321656: putative Triosephosphate isomerase, TC191823: myo-inositol 1-phosphate synthase, TC283096: heat shock protein 26. (CC:Cajete criollo, M21: Michoacán 21). (5.08 MB TIF) [file pone.0007531.s001.tif]
