# Supplementary material for: Analysis of Gene Expression and Physiological Responses in Three Mexican Maize Landraces under Drought Stress and Recovery Irrigation
Source: PLoS One. 2009 Oct 30;4(10):e7531. doi: 10.1371/journal.pone.0007531 (PMC2766256; doi:10.1371/journal.pone.0007531)
Supplement: Table S4 — BioMaps analysis of the common up-regulated genes among the three maize landraces at 17 days stress (0.04 MB DOC) [file pone.0007531.s005.doc]

**Table S4**. BioMaps analysis of the common up-regulated genes among the three maize landraces at 17 days stress

| **Term** | **Observed frequency** | **Expected Frequency** | **P-value** |
| --- | --- | --- | --- |
| **Cellular sensing and response to external stimulus** | 25 genes, 20.7% | 4.7% | 3.81E-08 |
| **INTERACTION WITH THE ENVIRONMENT** | 25 genes, 20.7% | 5.3% | 3.92E-07 |
| **Chemoperception and response** | 18 genes, 14.9% | 2.8% | 8.16E-07 |
| **Cell wall** | 9 genes,7.4% | 0.9% | 0.00019 |
| **Stress response** | 16 genes, 13.2% | 2.9% | 4.48E-05 |
| **Temperature perception and response** | 9 genes, 7.4% | 0.9% | 0.00016 |
| **CELL RESCUE, DEFENSE AND VIRULENCE** | 20 genes, 16.5% | 4.9% | 0.00019 |
| **Heat shock response** | 6 genes, 5% | 0.3% | 0.00021 |
| **Plant hormonal regulation** | 12 genes, 9.9% | 2% | 0.00049 |
| **SYSTEMIC INTERACTION WITH THE ENVIRONMENT** | 13 genes, 10.7% | 2.4% | 0.0008 |
| **Fruit development and ripening** | 7 genes, 5.8% | 0.6% | 0.00149 |
| **Extracellular matrix component** | 5 genes, 4.1% | 0.3% | 0.00669 |
| **Other plant signalling molecules response (jasmonic acid, salicylic acid etc.)** | 6 genes, 5% | 0.6% | 0.01214 |
| **BIOGENESIS OF CELLULAR COMPONENTS** | 18 genes, 14.9% | 5.5% | 0.01215 |
| **Plant development** | 11 genes, 9.1% | 2.4% | 0.01895 |
| **Regulator of G-proteins signalling** | 2 genes, 1.7% | 0% | 0.04163 |
