# Supplementary material for: Analysis of Gene Expression and Physiological Responses in Three Mexican Maize Landraces under Drought Stress and Recovery Irrigation
Source: PLoS One. 2009 Oct 30;4(10):e7531. doi: 10.1371/journal.pone.0007531 (PMC2766256; doi:10.1371/journal.pone.0007531)
Supplement: Table S5 — BioMaps analysis of the common down-regulated genes among the three maize landraces at 17 days stress (0.03 MB DOC) [file pone.0007531.s006.doc]

**Table S5. BioMaps analysis of the common down-regulated genes among the three maize landraces at 17 days stress**

| **Term** | **Observed frequency** | **Expected Frequency** | **P-value** |
| --- | --- | --- | --- |
| **Energy** | 10 genes, 14.9% | 1.5% | 5.14E-06 |
| **Photosynthesis** | 5 genes, 7.5% | 0.2% | 1.73E-05 |
| **Energy conversion and regeneration** | 4 genes, 6% | 0.2% | 0.0014 |
| **CELLULAR TRANSPORT, TRANSPORT FACILITATION AND TRANSPORT ROUTES** | 17 genes, 25.4% | 8.6% | 0.00322 |
| **Tetraterpene metabolism** | 3 genes, 4.5% | 0.1% | 0.00354 |
