# Supplementary material for: Analysis of Gene Expression and Physiological Responses in Three Mexican Maize Landraces under Drought Stress and Recovery Irrigation
Source: PLoS One. 2009 Oct 30;4(10):e7531. doi: 10.1371/journal.pone.0007531 (PMC2766256; doi:10.1371/journal.pone.0007531)
Supplement: Table S6 — BioMaps analysis of the up-regulated genes common in the tolerant landraces at 17 days stress. (0.04 MB DOC) [file pone.0007531.s007.doc]

**Table S6. BioMaps analysis of the up-regulated genes common in the tolerant landraces at 17 days stress**

| **Term** | **Observed frequency** | **Expected Frequency** | **P-value** |
| --- | --- | --- | --- |
| **Stress response** | 31 genes, 15.3% | 2.9% | 5.39E-12 |
| **Cellular sensing and response to external stimulus** | 39 genes, 19.2% | 4.7% | 8.38E-12 |
| **INTERACTION WITH THE ENVIRONMENT** | 39 genes, 19.2% | 5.3% | 2.96E-10 |
| **CELL RESCUE, DEFENSE AND VIRULENCE** | 37 genes, 18.2% | 4.9% | 7.45E-10 |
| **Heat shock response** | 11 genes, 5.4% | 0.3% | 5.30E-09 |
| **Temperature perception and response** | 16 genes, 7.9% | 0.9% | 8.42E-09 |
| **Chemoperception and response** | 24 genes, 11.8% | 2.8% | 4.64E-07 |
| **Cell wall** | 16 genes, 7.9% | 1.6% | 2.15E-05 |
| **SYSTEMIC INTERACTION WITH THE ENVIRONMENT** | 19 genes, 9.4% | 2.4% | 8.41E-05 |
| **Plant development** | 19 genes, 9.4% | 2.4% | 9.00E-05 |
| **Fruit development and ripening** | 9 genes, 4.4% | 0.6% | 0.00111 |
| **CELL FATE** | 12 genes, 5.9% | 1.4% | 0.00406 |
| **Glyoxylate cycle**  **Plant hormonal regulation**  **Regulation of directional cell growth** | 3 genes, 1.5%  14 genes, 6.9%  2 genes, 1% | 0%  2%  0% | 0.00684  0.0077  0.02422 |
