# Supplementary material for: Analysis of Gene Expression and Physiological Responses in Three Mexican Maize Landraces under Drought Stress and Recovery Irrigation
Source: PLoS One. 2009 Oct 30;4(10):e7531. doi: 10.1371/journal.pone.0007531 (PMC2766256; doi:10.1371/journal.pone.0007531)
Supplement: Table S7 — BioMaps analysis of the down-regulated genes common in the tolerant landraces at 17 days stress. (0.04 MB DOC) [file pone.0007531.s008.doc]

**Table S7. BioMaps analysis of the down-regulated genes common in the tolerant landraces at 17 days stress**

| **Term** | **Observed frequency** | **Expected Frequency** | **P-value** |
| --- | --- | --- | --- |
| **Energy** | 22 genes, 12% | 1.5% | 1.06E-11 |
| **Photosynthesis** | 9 genes, 4.9% | 0.2% | 1.23E-08 |
| **Energy conversion and regeneration** | 7 genes, 3.8% | 0.2% | 2.97E-05 |
| **Metabolism** | 59 genes, 32.2% | 17.6% | 0.00015 |
| **Cell rescue, defense and virulence** | 25 genes, 13.7% | 4.9% | 0.00056 |
| **Plastid** | 44 genes, 24% | 12.3% | 0.00117 |
| **Cellular transport, transport facilitation and transport routes** | 34 genes, 18.6% | 8.6% | 0.00226 |
| **C-compound and carbohydrate transport** | 7 genes, 3.8% | 0.5 % | 0.0042 |
| **C-3 compound metabolism** | 6 genes, 3.3% | 0.3% | 8.41E-05 |
| **Tetraterpene metabolism** | 4 genes, 2.2% | 0.1% | 0.00491 |
| **Chloroplast** | 42 genes, 23% | 12.2% | 0.00519 |
| **Sugar, glucoside, polyol and carboxylate catabolism** | 6 genes, 3.3% | 0.3% | 0.00539 |
| **C-compound and carbohydrate metabolism** | 24 genes, 13.1% | 5.9% | 0.03133 |
| **Nucleotide – sugar metabolism** | 7 genes, 3.8% | 0.7% | 0.03573 |
| **Metabolism of porphyrins** | 4 genes, 2.2% | 0.2% | 0.04211 |
| **Stress response** | 15 genes, 8.2% | 2.9% | 0.04216 |
