# Supplementary material for: Analysis of Gene Expression and Physiological Responses in Three Mexican Maize Landraces under Drought Stress and Recovery Irrigation
Source: PLoS One. 2009 Oct 30;4(10):e7531. doi: 10.1371/journal.pone.0007531 (PMC2766256; doi:10.1371/journal.pone.0007531)
Supplement: Table S8 — BioMaps analysis of the common up-regulated genes among the three maize landraces at recovery irrigation. (0.03 MB DOC) [file pone.0007531.s009.doc]

**Table S8. BioMaps analysis of the common up-regulated genes among the three maize landraces at recovery irrigation**

| **Term** | **Observed frequency** | **Expected Frequency** | **P-value** |
| --- | --- | --- | --- |
| **Plastid** | 84 genes, 29.3% | 12.3% | 2.06E-12 |
| **Chloroplast** | 83 genes, 28.9% | 12.2% | 4.24E-12 |
| **Photosynthesis** | 8 genes,2.8% | 0.2 % | 1.62E-05 |
| **METABOLISM** | 87 genes, 30.3% | 17.6% | 1.63E-05 |
| **ENERGY** | 18 genes, 6.3% | 1.5% | 8.18E-05 |
| **SUBCELLULAR LOCALIZATION** | 144 genes, 50.2% | 37.6% | 0.00171 |
| **Energy conversion and regeneration** | 6 genes, 2.1% | 0.2% | 0.00939 |
| **CELLULAR TRANSPORT, TRANSPORT FACILITATION AND TRANSPORT ROUTES** | 45 genes, 15.7% | 8.6% | 0.01243 |
| **Secondary metabolism** | 14 genes, 4.9% | 1.5% | 0.02147 |
| **Metabolism of porphyrins** | 5 genes, 1.7% | 0.2% | 0.02637 |
