# Supplementary material for: Analysis of Gene Expression and Physiological Responses in Three Mexican Maize Landraces under Drought Stress and Recovery Irrigation
Source: PLoS One. 2009 Oct 30;4(10):e7531. doi: 10.1371/journal.pone.0007531 (PMC2766256; doi:10.1371/journal.pone.0007531)
Supplement: Table S9 — BioMaps analysis of the common down-regulated genes among the three maize landraces at recovery irrigation. (0.04 MB DOC) [file pone.0007531.s010.doc]

**Table S9. BioMaps analysis of the common down-regulated genes among the three maize landraces at recovery irrigation**

| **Term** | **Observed frequency** | **Expected Frequency** | **P-value** |
| --- | --- | --- | --- |
| **Stress response** | 49 genes, 14.3% | 2.9% | 3.70E-18 |
| **CELL RESCUE, DEFENSE AND VIRULENCE** | 59 genes, 17.3% | 4.9% | 6.39E-15 |
| **Cellular sensing and response to o external stimulus** | 57 genes,16.7% | 4.7% | 1.48E-14 |
| **INTERACTION WITH THE ENVIRONMENT** | 58 genes, 17% | 5.3% | 5.64E-13 |
| **Heat shock response** | 16 genes, 4.7% | 0.3% | 1.52E-12 |
| **Temperature perception and response** | 22 genes, 6.4% | 0.9% | 1.43E-10 |
| **Chemoperception and response** | 37 genes, 10.8% | 2.8% | 4.74E-10 |
| **Water response** | 13 genes, 3.8% | 0.4% | 4.33E-07 |
| **C-compound and carbohydrate metabolism** | 50 genes, 14.6% | 5.9% | 6.35E-07 |
| **METABOLISM** | 104 genes, 30.4% | 17.6% | 7.80E-07 |
| **Abscisic acid response** | 13 genes, 3.8% | 0.6% | 1.69E-05 |
| **Osmotic and salt stress response** | 13 genes, 3.8% | 0.7% | 0.00028 |
| **SYSTEMIC INTERACTION WITH THE ENVIRONMENT** | 24 genes, 7% | 2.4% | 0.00067 |
| **Calcium binding** | 11 genes, 3.2% | 0.6% | 0.00216 |
| **Plant hormonal regulation** | 20 genes, 5.8% | 2% | 0.00296 |
| **Second messenger mediated signal transduction** | 15 genes, 4.4% | 1.2% | 0.00323 |
| **Plant / fungal specific systemic sensing and response** | 21 genes, 6.1% | 2.2% | 0.00433 |
| **Hormone mediated signal transduction** | 11 genes, 3.2% | 0.7% | 0.00494 |
| **Cell wall** | 12 genes, 3.5% | 0.9% | 0.01581 |
| **CELL FATE** | 15 genes, 4.4% | 1.4% | 0.01677 |
| **Cell wall** | 16 genes, 4.7% | 1.6% | 0.02019 |
| **Oxidative stress response** | 10 genes, 2.9% | 0.7% | 0.03395 |
| **Glyoxylate cycle** | 3 genes, 0.9% | 0% | 0.03974 |
