# Supplementary material for: Analysis of Gene Expression and Physiological Responses in Three Mexican Maize Landraces under Drought Stress and Recovery Irrigation
Source: PLoS One. 2009 Oct 30;4(10):e7531. doi: 10.1371/journal.pone.0007531 (PMC2766256; doi:10.1371/journal.pone.0007531)
Supplement: Table S10 — BioMaps analysis of the up-regulated genes common in the tolerant landraces at recovery irrigation. (0.06 MB DOC) [file pone.0007531.s011.doc]

Table S10. BioMaps analysis of the up-regulated genes common in the tolerant landraces at recovery irrigation

| **Term** | **Observed frequency** | **Expected Frequency** | **P-value** |
| --- | --- | --- | --- |
| **Unannotated** | 2 genes, 0.3% | 0% | 0 |
| **Plastid** | 201 genes, 29% | 12.3% | 3.08E-30 |
| **Chloroplast** | 197 genes, 28.4% | 12.2% | 1.00E-28 |
| **SUBCELLULAR LOCALIZATION** | 380 genes, 54.8% | 37.6% | 4.06E-18 |
| **METABOLISM** | 215 genes,31% | 17.6% | 6.93E-16 |
| **ENERGY** | 47 genes, 6.8% | 1.5% | 2.28E-15 |
| **Photosynthesis** | 17 genes, 2.4% | 0.2% | 3.04E-12 |
| **Metabolism of porphyrins** | 16 genes, 2.3% | 0.2% | 6.65E-12 |
| **Metabolism of secondary products derived from glycine, L-serine and L-alanine** | 16 genes, 2.3% | 0.2% | 1.86E-10 |
| **C-compound and carbohydrate metabolism** | 85 genes, 12.2% | 5.9% | 4.7E-08 |
| **Light absorption** | 9 genes, 1.3% | 0.1% | 4.7E-08 |
| **Secondary metabolism** | 32 genes, 4.6% | 1.5% | 5.65E-06 |
| **Sugar, glucoside, polyol and carboxylate metabolism** | 50 genes, 7.2% | 3.1% | 8.01E-06 |
| **Energy conversion and regeneration** | 12 genes, 1.7% | 0.2% | 1.37E-05 |
| **Photoperception and response** | 21 genes, 3% | 0.9% | 0.0003 |
| **Biosynthesis of vitamins, cofactors, and prosthetic** | 14 genes, 2% | 0.4% | 0.00078 |
| **Transported compounds (substrates)** | 79 genes, 11.4% | 6.7% | 0.00085 |
| **Sugar, glucoside, polyol and carboxylate catabolism** | 12 genes, 1.7% | 0.3% | 0.00107 |
| **Cytoplasm** | 51 genes, 7.3% | 3.8% | 0.00245 |
| **Complex cofactor/cosubstrate/vitamine binding** | 13 genes, 1.9% | 0.4% | 0.00254 |
| **Cellular sensing and response to external stimulus** | 59 genes, 8.5% | 4.7% | 0.00272 |
| **CELLULAR TRANSPORT, TRANSPORT FACILITATION** | 93 genes, 13.4% | 8.6% | 0.00385 |
| **Glycolysis and gluconeogenesis** | 10 genes, 1.4% | 0.3% | 0.00402 |
| **Plastid** | 8 genes, 1.2% | 0.2% | 0.00412 |
| **Metabolism of glutamate** | 7 genes, 1% | 0.1% | 0.0052 |
| **Chromoplast** | 6 genes, 0.9% | 0.1% | 0.00823 |
| **INTERACTION WITH THE ENVIRONMENT** | 62 genes, 8.9% | 5.3% | 0.01119 |
| **Tetraterpenes (carotenoids) metabolism** | 6 genes, 0.9% | 0.1% | 0.01643 |
| **Temperature perception and response** | 18 genes, 2.6% | 0.9% | 0.01811 |
| **Electron transport** | 35 genes, 5% | 2.5% | 0.0185 |
| **C-3 compound metabolism** | 10 genes, 1.4% | 0.3% | 0.02295 |
| **Amino acid metabolism** | 21 genes, 3% | 1.2% | 0.03802 |
| **Isoprenoid metabolism** | 14 genes, 2% | 0.6% | 0.03937 |
| **Pentose-phosphate pathway** | 7 genes, 1% | 0.2% | 0.03984 |
| **Metabolism of vitamins, cofactors, and prosthetic groups** | 17 genes, 2.4% | 0.9% | 0.04692 |
