# Supplementary material for: Analysis of Gene Expression and Physiological Responses in Three Mexican Maize Landraces under Drought Stress and Recovery Irrigation
Source: PLoS One. 2009 Oct 30;4(10):e7531. doi: 10.1371/journal.pone.0007531 (PMC2766256; doi:10.1371/journal.pone.0007531)
Supplement: Table S11 — BioMaps analysis of the down-regulated genes common in the tolerant landraces at recovery irrigation. (0.05 MB DOC) [file pone.0007531.s012.doc]

**Table S11. BioMaps analysis of the down-regulated genes common in the tolerant landraces at recovery irrigation**

| **Term** | **Observed frequency** | **Expected Frequency** | **P-value** |
| --- | --- | --- | --- |
| **Unannotated** | 3 genes, 0.5% | 0% | 0 |
| **Stress response** | 66 genes, 10.4% | 2.9% | 4.35E-17 |
| **Cellular sensing and response to external stimulus** | 81 genes, 12.8% | 4.7 % | 6.91E-14 |
| **CELL RESCUE, DEFENSE AND VIRULENCE** | 82 genes, 13% | 4.9% | 2.87E-13 |
| **INTERACTION WITH THE ENVIRONMENT** | 83 genes, 13.1% | 5.3% | 4.57E-12 |
| **Heat shock response** | 18 genes, 2.8% | 0.3% | 1.77E-10 |
| **Temperature perception and response** | 29 genes, 4.6% | 0.9% | 1.99E-10 |
| **METABOLISM** | 183 genes, 29% | 17.6% | 2.16E-10 |
| **Chemoperception and response** | 15 genes, 2.4% | 0.7% | 1.216E-09 |
| **C-compound and carbohydrate metabolism** | 83 genes, 13.1% | 5.9% | 2.02E-09 |
| **Osmotic and salt stress response** | 21 genes, 3.3% | 0.7% | 2.23E-06 |
| **Water response** | 16 genes, 2.5% | 0.4% | 2.55E-06 |
| **Abscisic acid response** | 16 genes, 2.5% | 0.6% | 0.00018 |
| **Glyoxylate cycle** | 5 genes, 0.8% | 0% | 0.00035 |
| **SYSTEMIC INTERACTION WITH THE ENVIRONMENT** | 35 genes, 5.5% | 2.4% | 0.00141 |
| **CELLULAR TRANSPORT, TRANSPORT FACILITATION AND** | 87 genes, 13.8% | 8.6% | 0.00232 |
| **Oxidative stress response** | 16 genes, 2.5% | 0.7% | 0.00328 |
| **Complex cofactor/cosubstrate/vitamine binding** | 12 genes, 1.9% | 0.4% | 0.00449 |
| **REGULATION OF METABOLISM AND PROTEIN FUNCTION** | 31 genes, 4.9% | 2.2% | 0.00877 |
| **Cold shock response** | 13 genes, 2.1% | 0.6% | 0.01477 |
| **Cell aging** | 7 genes, 1.1% | 0.2% | 0.01488 |
| **Plant hormonal regulation** | 28 genes, 4.4% | 2% | 0.01508 |
| **Plant / fungal specific systemic sensing and response** | 30 genes, 4.7% | 2.2% | 0.01663 |
| **CELL FATE** | 22 genes, 3.5% | 1.4% | 0.01889 |
| **Hormone mediated signal transduction** | 14 genes, 2.2% | 0.7% | 0.03202 |
| **Transported compounds (substrates)** | 67 genes, 10.6% | 6.7% | 0.03706 |
| **NAD/NADP binding** | 7 genes, 1.1% | 0.2% | 0.03914 |
| **Calcium binding** | 13 genes, 2.1% | 0.6% | 0.04791 |
| **Response to biotic stimulus** | 15 genes, 2.4% | 0.8% | 0.04906 |
